# Supplementary material for: Interrogation of enhancer function by enhancer-targeting CRISPR epigenetic editing
Source: Nat Commun. 2020 Jan 24;11:485. doi: 10.1038/s41467-020-14362-5 (PMC6981169; doi:10.1038/s41467-020-14362-5)
Supplement: Supplementary file 1 — Supplementary Information [file 41467_2020_14362_MOESM1_ESM.pdf]

Supplementary Information

Interrogation of Enhancer Function by Enhancer-Targeting CRISPR Epigenetic Editing

Li and Liu et al.

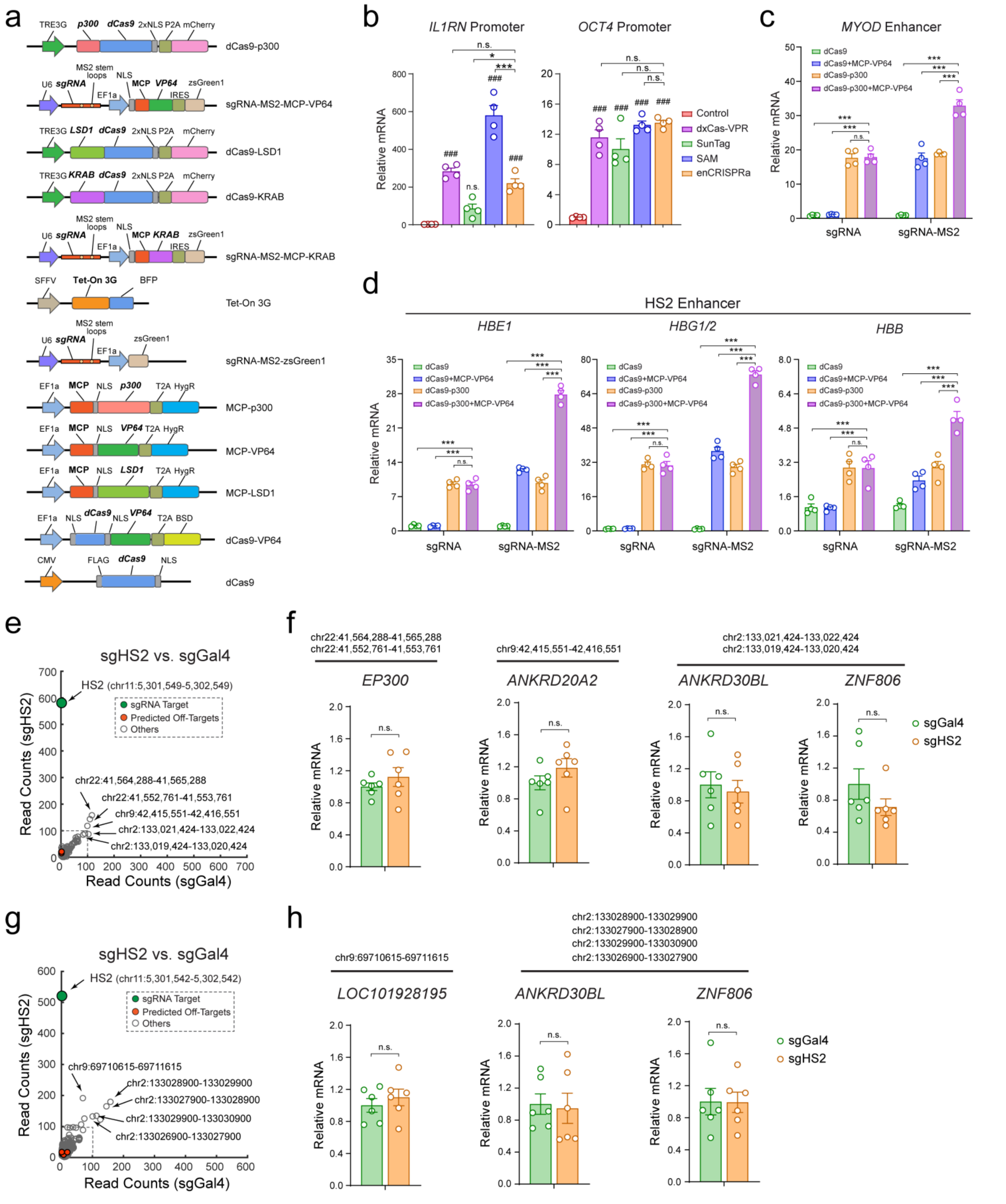

Supplementary Figure 1. Evaluation of enCRISPRa in Target Gene Activation

(a) Schematic of the constructs used in this study.

(b) Expression of *IL1RN* and *OCT4* upon dxCas9-VPR, SunTag, SAM or enCRISPRa-mediated promoter activation in HEK293T cells. mRNA expression relative to non-transduced cells is shown as mean ± SEM (N = 4 experiments). The differences between control and dCas9 activators were analyzed by a one-way ANOVA. $^{\#}P < 0.05$, $^{\#\#}P < 0.01$, $^{\#\#\#}P <$

0.001. The difference between enCRISPRa and other dCas9 activators were analyzed by a one-way ANOVA. $*P < 0.05$, $**P < 0.01$, $***P < 0.001$, n.s. not significant.

(c) Expression of *MYOD* upon dCas9 alone, dCas9 + MCP-VP64, dCas9-p300, or dCas9-p300 + MCP-VP64 mediated enhancer activation together with the *MYOD* enhancer-targeting sgRNA with or with MS2 loops in HEK293T cells. Results are mean ± SEM ($N = 4$ experiments). The differences between different groups were analyzed by a two-way ANOVA. $***P < 0.001$, n.s. not significant.

(d) Expression of *HBE1*, *HBG1/2* and *HBB* upon dCas9 alone, dCas9 + MCP-VP64, dCas9-p300, or dCas9-p300 + MCP-VP64 mediated enhancer activation with the HS2 enhancer-targeting sgRNA with or with MS2 in HEK293T cells. Results are mean ± SEM ($N = 4$ experiments) and analyzed by a two-way ANOVA. $***P < 0.001$, n.s. not significant.

(e) Genome-wide analysis of dCas9 binding in HEK293T cells expressing sgHS2 or sgGal4. The same plot as Fig. 1f is shown except that the top 5 potential dCas9 off-targets are labeled.

(f) Expression of the nearest neighbor genes of the top 5 potential dCas9 off-targets in HEK293T cells. Results are mean ± SEM ($N = 6$ experiments) and analyzed by a two-sided *t*-test. n.s. not significant.

(g) Genome-wide analysis of dCas9 binding in K562 cells expressing sgHS2 or sgGal4. The same plot as Fig. 2d is shown except that the top 5 potential dCas9 off-targets are labeled.

(h) Expression of the nearest neighbor genes of the top 5 potential dCas9 off-targets in K562 cells. Results are mean ± SEM ($N = 6$ experiments) and analyzed by a two-sided *t*-test. Source data are provided as a Source Data file.

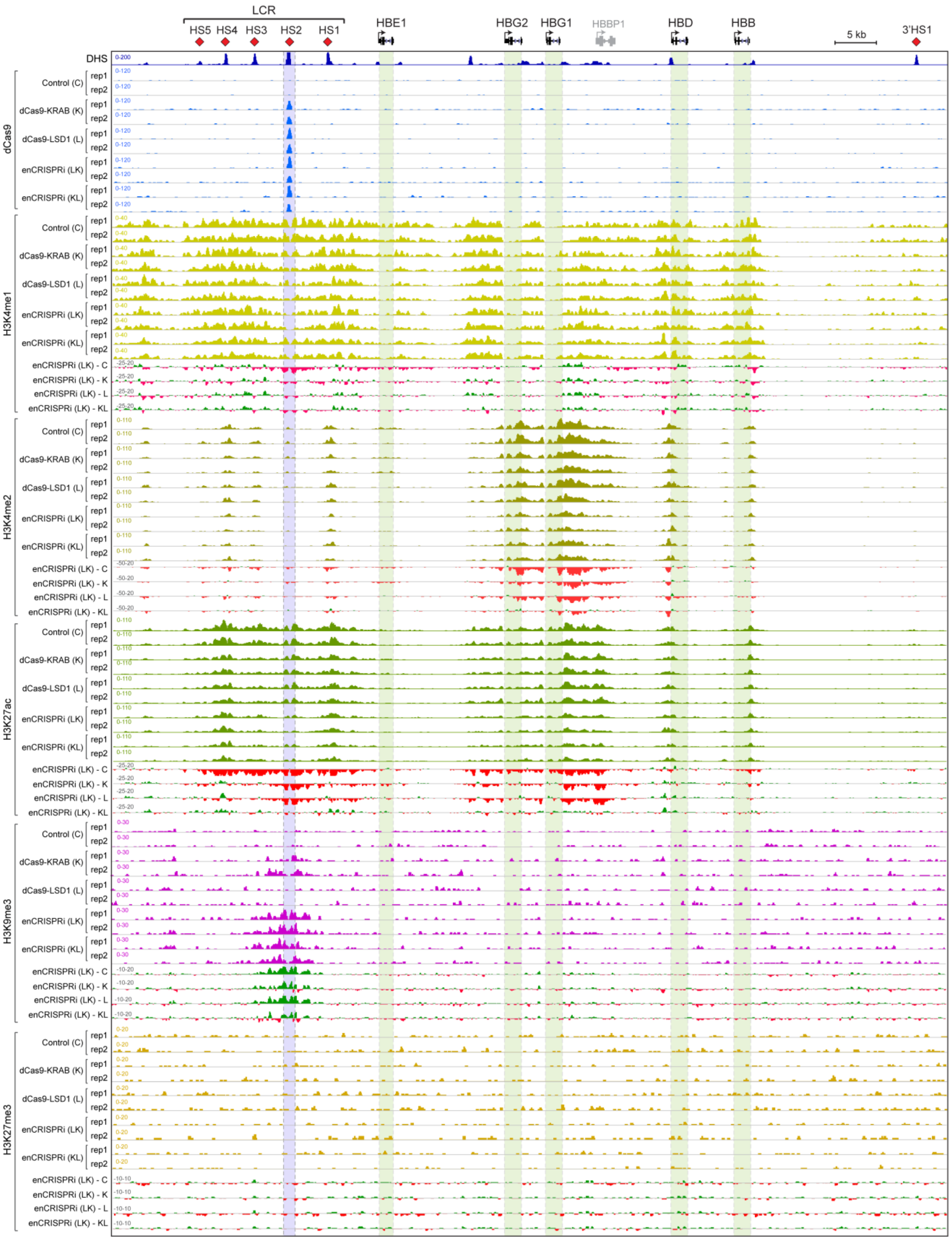

Supplementary Figure 2. enCRISPRi Leads to Locus-Wide Epigenetic Reprogramming

Similar to Fig. 3, density maps are shown for ChIP-seq of dCas9, H3K4me1, H3K4me2, H3K27ac, H3K9me3 and H3K27me3 at the β-globin cluster (chr11: 5,222,500-5,323,700; hg19) in K562 cells co-expressing non-targeting sgGal4 (control or C) or sgHS2 with dCas9-KRAB (K), dCas9-LSD1 (L) or enCRISPRi (LK and KL). Regions showing increased or decreased ChIP-seq signals in enCRISPRi (LK) relative to control, dCas9-KRAB, dCas9-LSD1 or enCRISPRi (KL) (enCRISPRi – C, enCRISPRi – K, enCRISPRi – L, or enCRISPRi – KL) are depicted in *green* and *red*, respectively. Blue bars denote the sgRNA-targeted HS2 enhancer. Green bars denote the β-globin genes. Independent replicates (rep1 and rep2) are shown for all ChIP-seq experiments.

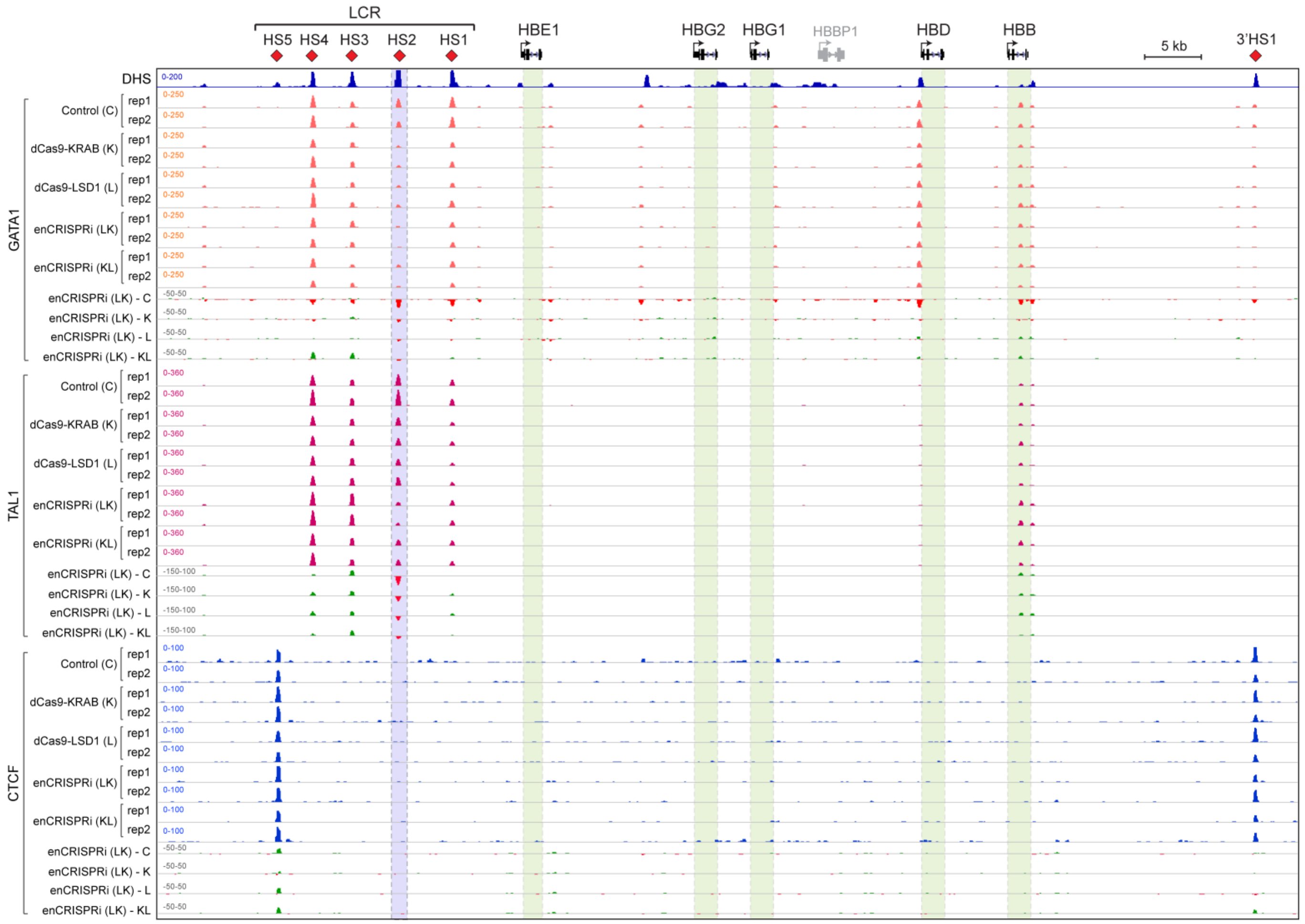

Supplementary Figure 3. Targeting enCRISPRi to the β-Globin Gene Cluster Interferes with TF Binding
Similar to Fig. 3, density maps are shown for ChIP-seq of GATA1, TAL1, and CTCF at the β-globin cluster (chr11: 5,222,500-5,323,700; hg19) in K562 cells. Independent ChIP-seq replicates (rep1 and rep2) are shown.

a

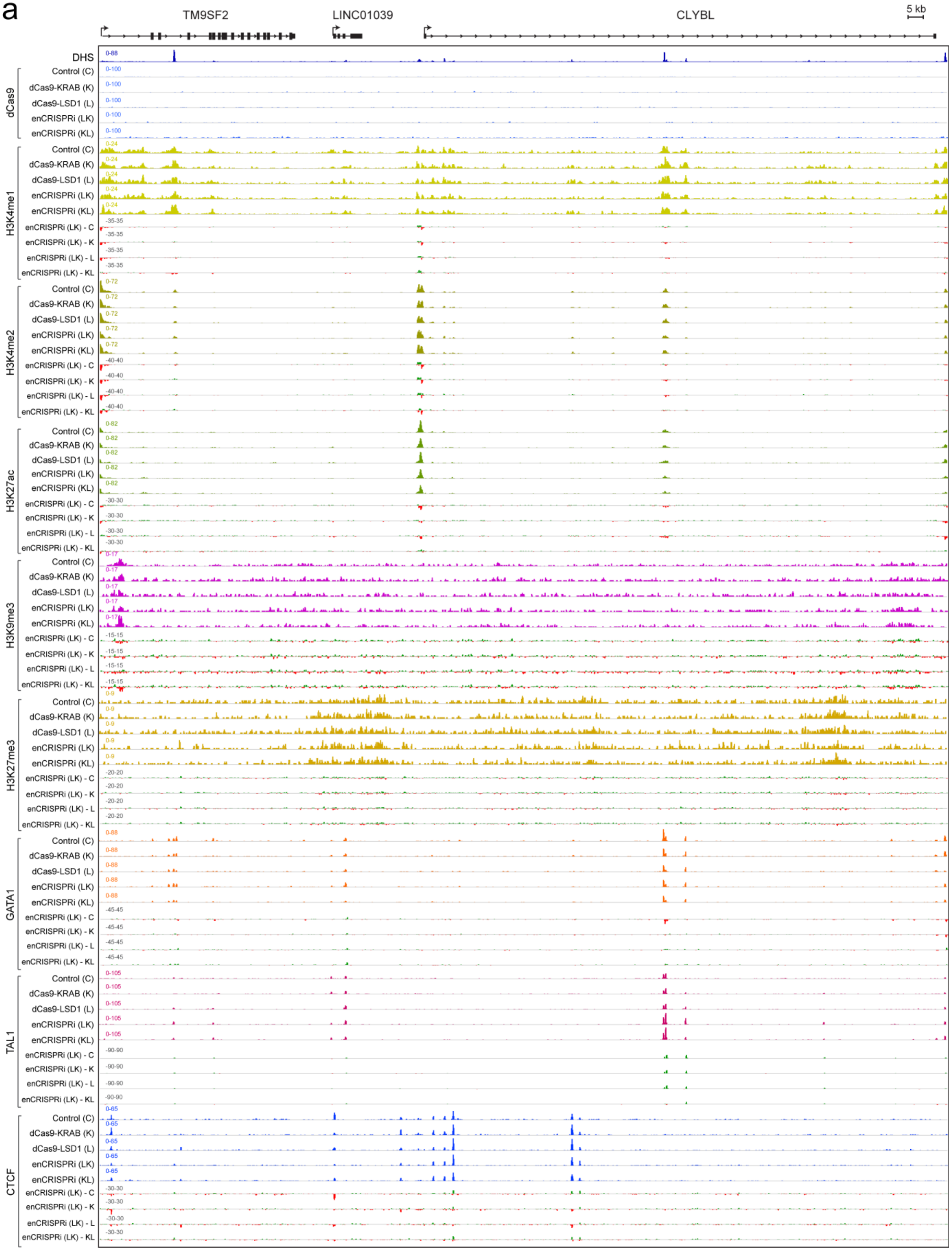

TM9SF2
LINC01039
CLYBL
5 kb
DHS
dCas9
H3K4me1
H3K4me2
H3K27ac
H3K9me3
H3K27me3
GATA1
TAL1
CTCF
Control (C)
dCas9-KRAB (K)
dCas9-LSD1 (L)
enCRISPRi (LK)
enCRISPRi (KL)
enCRISPRi (LK) - C
enCRISPRi (LK) - K
enCRISPRi (LK) - L
enCRISPRi (LK) - KL

b

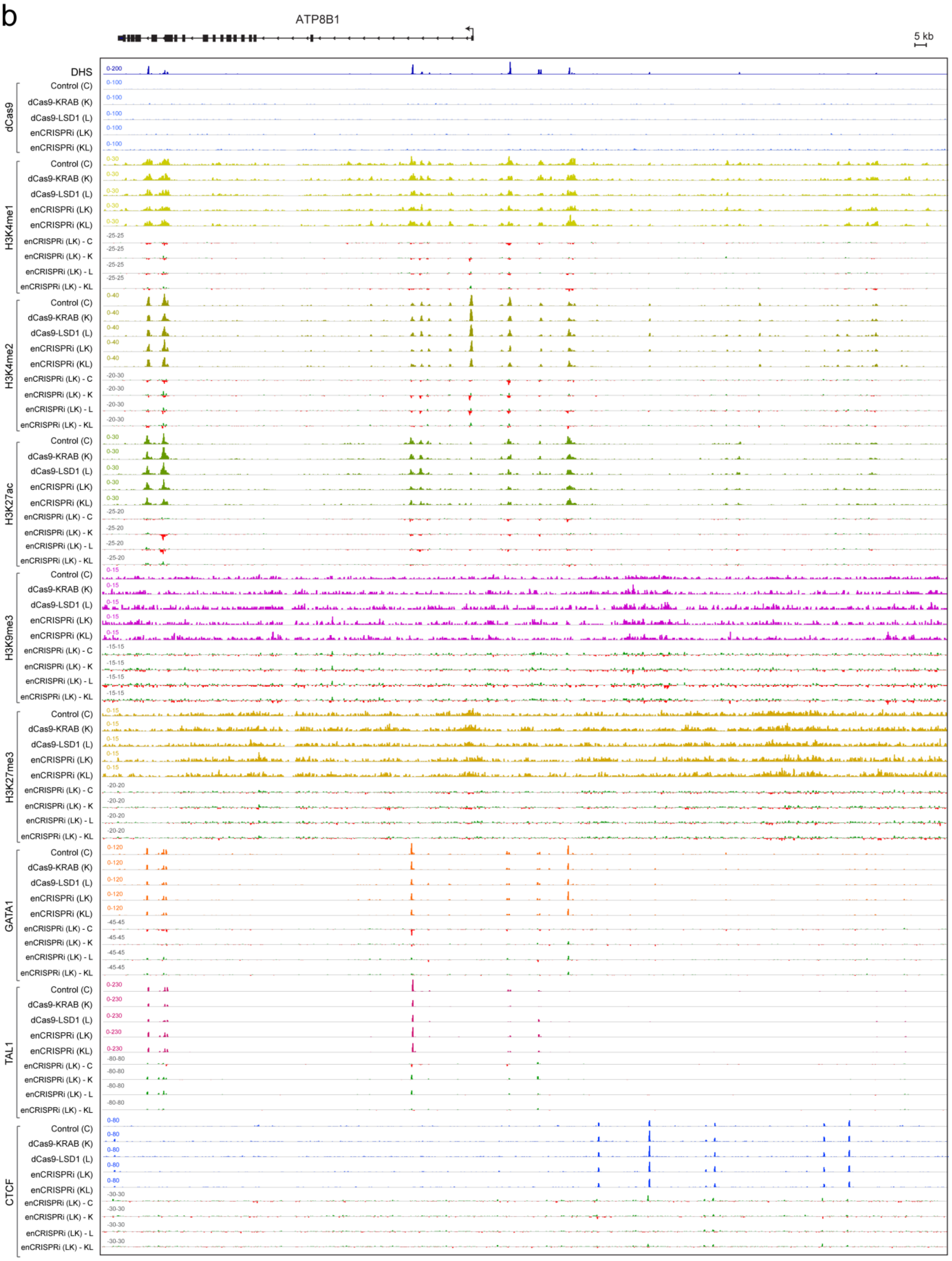

Supplementary Figure 4. Representative Non-Targeted Genomic Loci Upon HS2-Targeted enCRISPRi

(a) Similar to Fig. 3, density maps are shown for ChIP-seq of dCas9, H3K4me1, H3K4me2, H3K27ac, H3K9me3, H3K27me3, GATA1, TAL1, and CTCF at the *TM9SF2-LINC01039-CLYBL* locus (chr13: 100,154,607-100,430,801; hg19) in K562 cells.

(b) Similar to Fig. 3, density maps are shown for ChIP-seq of dCas9, H3K4me1, H3K4me2, H3K27ac, H3K9me3, H3K27me3, GATA1, TAL1, and CTCF at the *ATP8B1* locus (chr18: 55,292,001-55,668,879; hg19) in K562 cells.

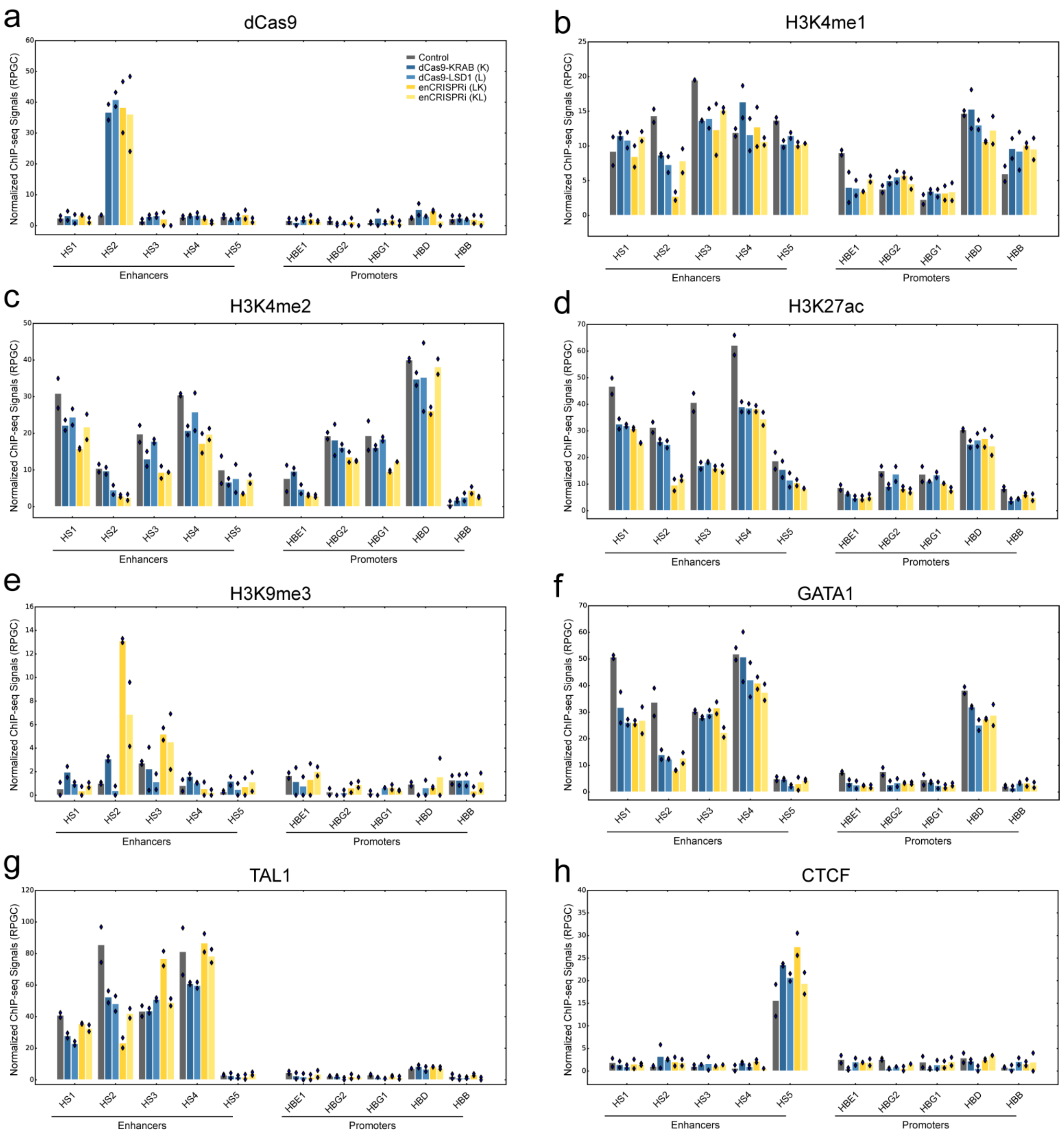

Supplementary Figure 5. Analysis of ChIP-seq Signals at the β-Globin Enhancers and Promoters

Bar graphs are shown for the normalized ChIP-seq signals as reads per genomic content (PRGC) at the β-globin enhancers (HS1 to HS5) and promoters (*HBE1*, *HBG2*, *HBG1*, *HBD* and *HBB*) for dCas9 (a), H3K4me1 (b), H3K4me2 (c), H3K27ac (d), H3K9me3 (e), GATA1 (f), TAL1 (g), and CTCF (h) in K562 cells co-expressing non-targeting sgGal4 (control) or sgHS2 with dCas9-KRAB (K), dCas9-LSD1 (L) or enCRISPRi (LK and KL). Dots indicate values from the independent ChIP-seq replicates (rep1 and rep2). Source data are provided as a Source Data file.

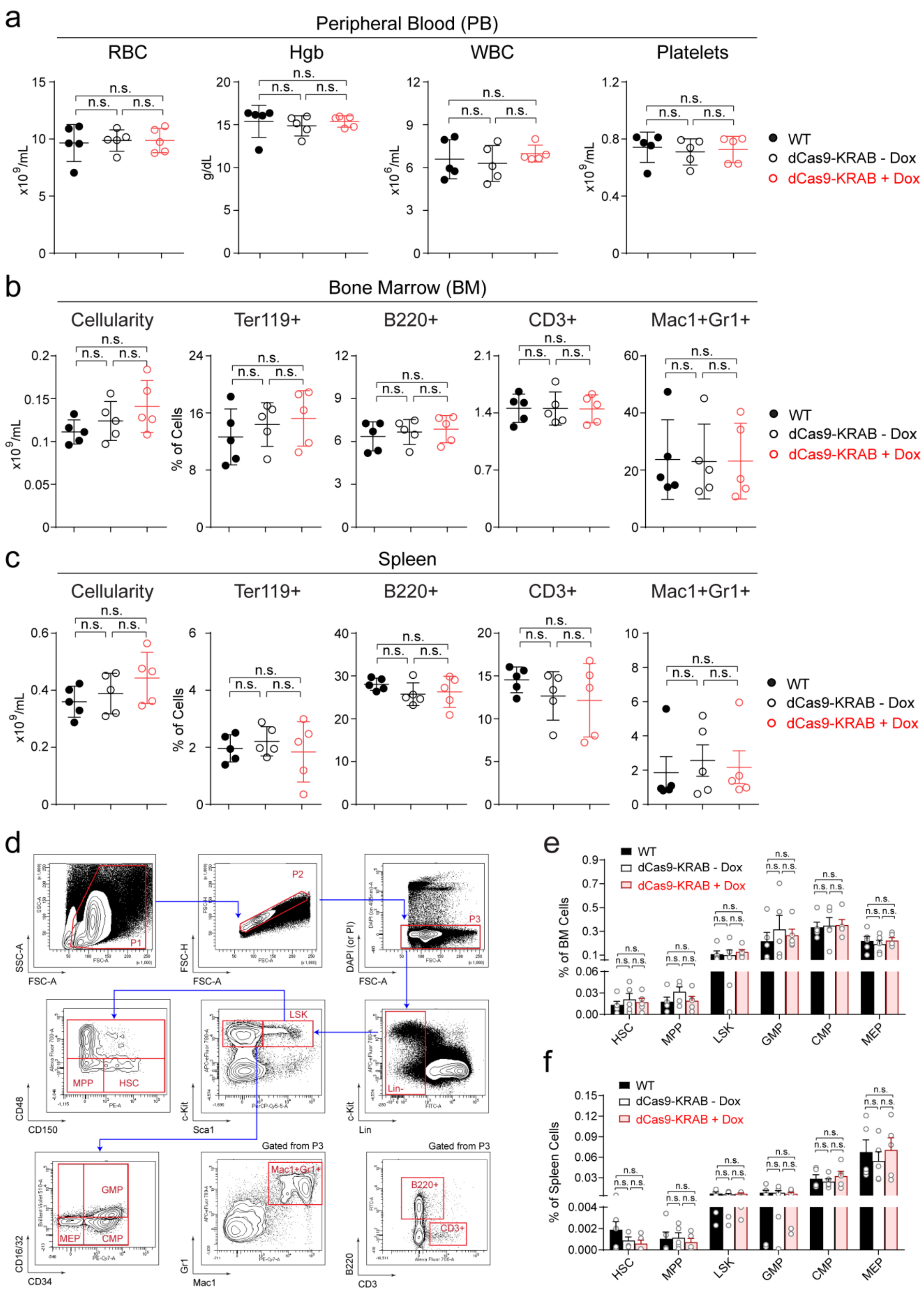

Supplementary Figure 6. Inducible dCas9-KRAB Expression Has No Effect on Hematopoietic Differentiation

(a) Complete blood count (CBC) of peripheral blood (PB) red blood cells (RBC), hemoglobin (Hgb), white blood cells (WBC) and platelets in mice 8 weeks after Dox treatment. N = 5 mice per group. Results are mean ± SD and analyzed by a repeated-measures one-way ANOVA with multiple comparisons. n.s. not significant.

(b) BM cellularity and frequencies of erythroid (Ter119$^+$), B-lymphoid (B220$^+$), T-lymphoid (CD3$^+$) and myeloid (Mac1$^+$Gr1$^+$) cells in mice 8 weeks after Dox treatment.

(c) Spleen cellularity and frequencies of erythroid, B-lymphoid, T-lymphoid and myeloid cells 8 weeks after Dox treatment.

(d) Representative flow cytometry gates are shown for the analysis of various hematopoietic stem/progenitor cells (HSPCs) and mature lineages in mouse bone marrow.

(e) Frequencies of HSPCs in BM 8 weeks after Dox treatment. *N* = 5 mice per group. Results are mean ± SEM and analyzed by a two-way ANOVA with multiple comparisons. n.s. not significant.

(f) Frequencies of HSPCs in spleen 8 weeks after Dox treatment. *N* = 5 mice per group. Source data are provided as a Source Data file.

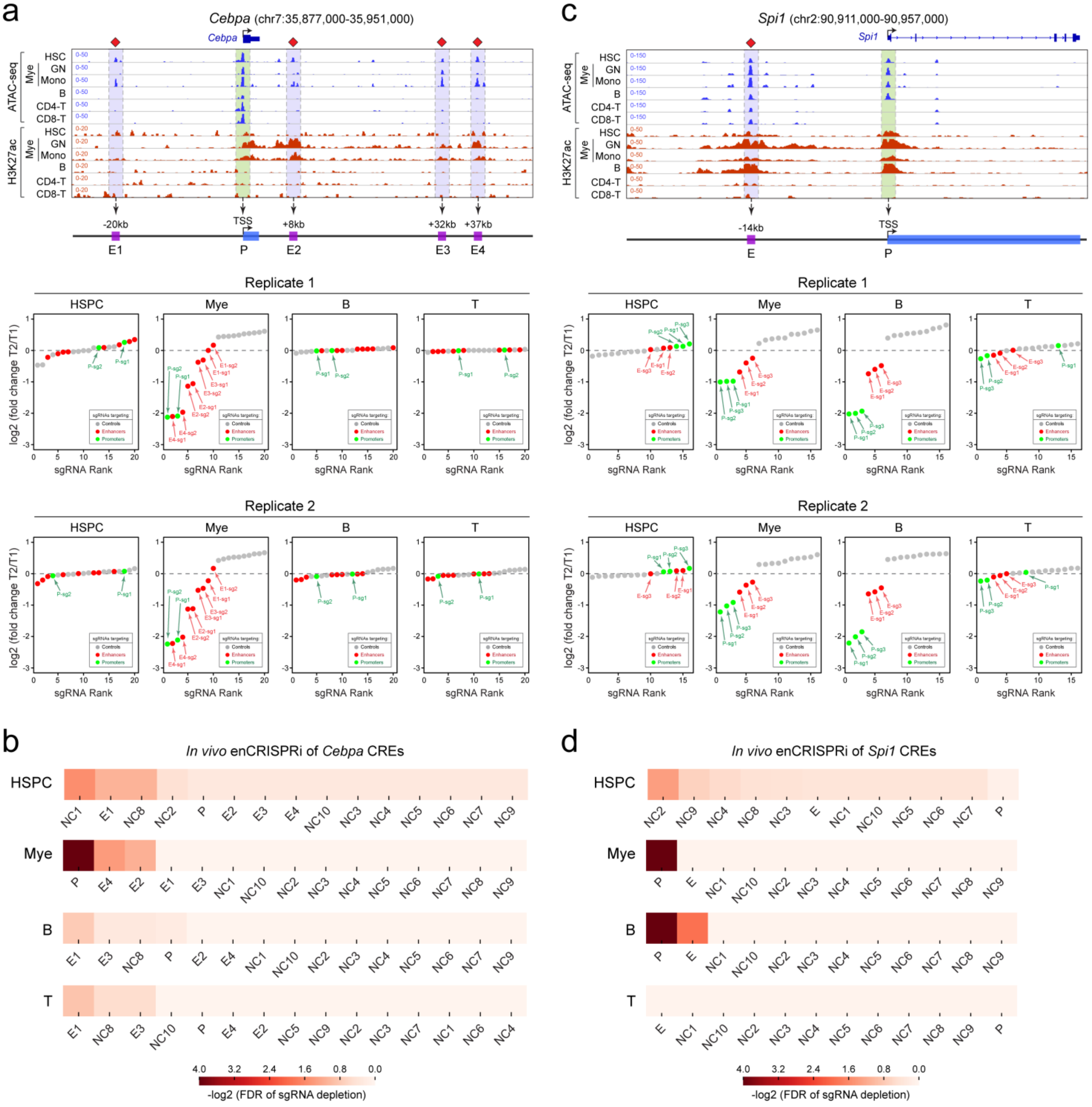

Supplementary Figure 7. Locus-Specific Enhancer Perturbation at *Cebpa* and *Spi1* Loci

(a) *In vivo* enCRISPRi perturbation of *Cebpa* CREs during hematopoiesis. Similar to Fig. 5e, waterfall plots are shown for target-specific sgRNAs (*green* and *red* dots) and non-targeting control sgRNAs (*grey* dots) by the normalized log2 fold changes in HSPCs, myeloid, T or B cells 16-weeks post-BMT (T2) relative to pooled sgRNA-transduced HSPCs (T1) in two independent replicate screens (*N* = 3 recipient mice per screen). The annotated *Cebpa* promoter (P) and enhancers (E1 to E4) are indicated by *green* and *blue* shaded lines.

(b) Ranking of the top depleted *Cebpa* CREs by the –log2 (FDR of sgRNA depletion). The significance (FDR) of depletion for each targeted enhancer, promoter or non-targeting control (NC) region in each cell type was calculated using the MAGECK test. P, promoter; E, enhancer; NC, non-targeting control.

(c) *In vivo* enCRISPRi perturbation of *Spi1* CREs during hematopoiesis. Similar to Fig. 5f, waterfall plots are shown for two independent replicate screens (*N* = 3 recipient mice per screen). The annotated *Spi1* promoter (P) and enhancer (E) are indicated by *green* and *blue* shaded lines.

(d) Ranking of the top depleted *Spi1* CREs by the –log2 (FDR of sgRNA depletion).

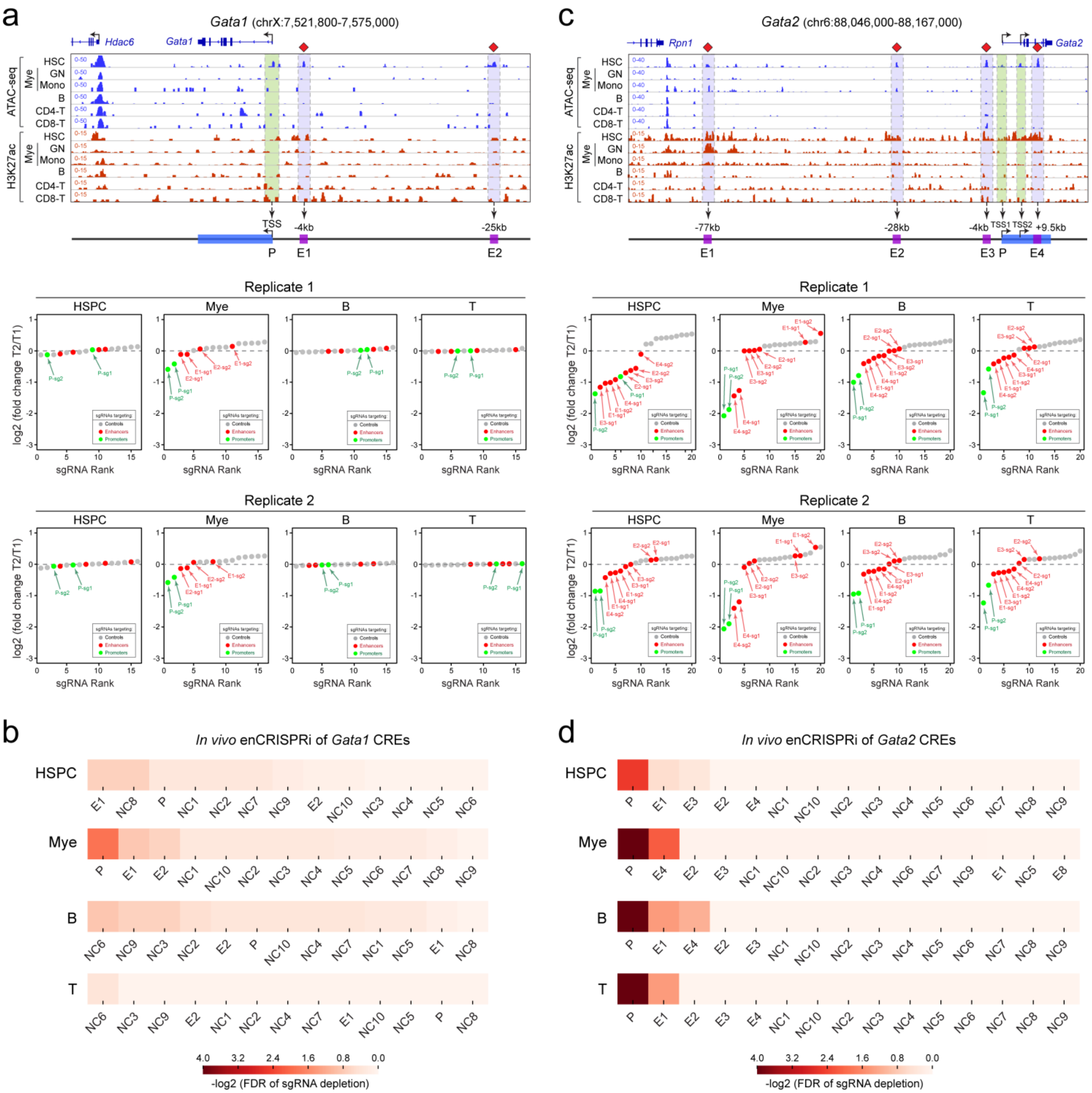

Supplementary Figure 8. Locus-Specific Enhancer Perturbation at *Gata1* and *Gata2* Loci

(a) *In vivo* enCRISPRi perturbation of *Gata1* CREs during hematopoiesis. Density maps are shown for ATAC-seq and H3K27ac ChIP-seq at the *Gata1* locus (chrX:7,521,800-7,575,000; mm9) in bone marrow HSC, granulocytes (GN), monocytes (Mono), B, CD4+ and CD8+ T cells, respectively. Waterfall plots are shown for target-specific sgRNAs (*green* and *red* dots) and non-targeting control sgRNAs (*grey* dots) by the normalized log2 fold changes in HSPCs, myeloid, T or B cells 16-weeks post-BMT (T2) relative to pooled sgRNA-transduced HSPCs (T1) in two independent replicate screens (*N* = 3 recipient mice per screen). The annotated *Gata1* promoter (P) and enhancers (E1 and E2) are indicated by *green* and *blue* shaded lines.

(b) Ranking of the top depleted *Gata1* CREs by the –log2 (FDR of sgRNA depletion). The significance (FDR) of depletion for each targeted enhancer, promoter or non-targeting control (NC) region in each cell type was calculated using the MAGECK test. P, promoter; E, enhancer; NC, non-targeting control.

(c) *In vivo* enCRISPRi perturbation of *Gata2* CREs during hematopoiesis. Density maps are shown for ATAC-seq and H3K27ac ChIP-seq at the *Gata2* locus (chr6:88,046,000-88,167,000; mm9) in bone marrow HSC, GN, Mono, B, CD4+ and CD8+ T cells, respectively. Waterfall plots are shown for two independent replicate screens (*N* = 3 recipient mice per screen). The annotated *Gata2* promoter (P) and enhancers (E1 to E4) are indicated by *green* and *blue* shaded lines.

(d) Ranking of the top depleted *Gata2* CREs by the –log2 (FDR of sgRNA depletion).

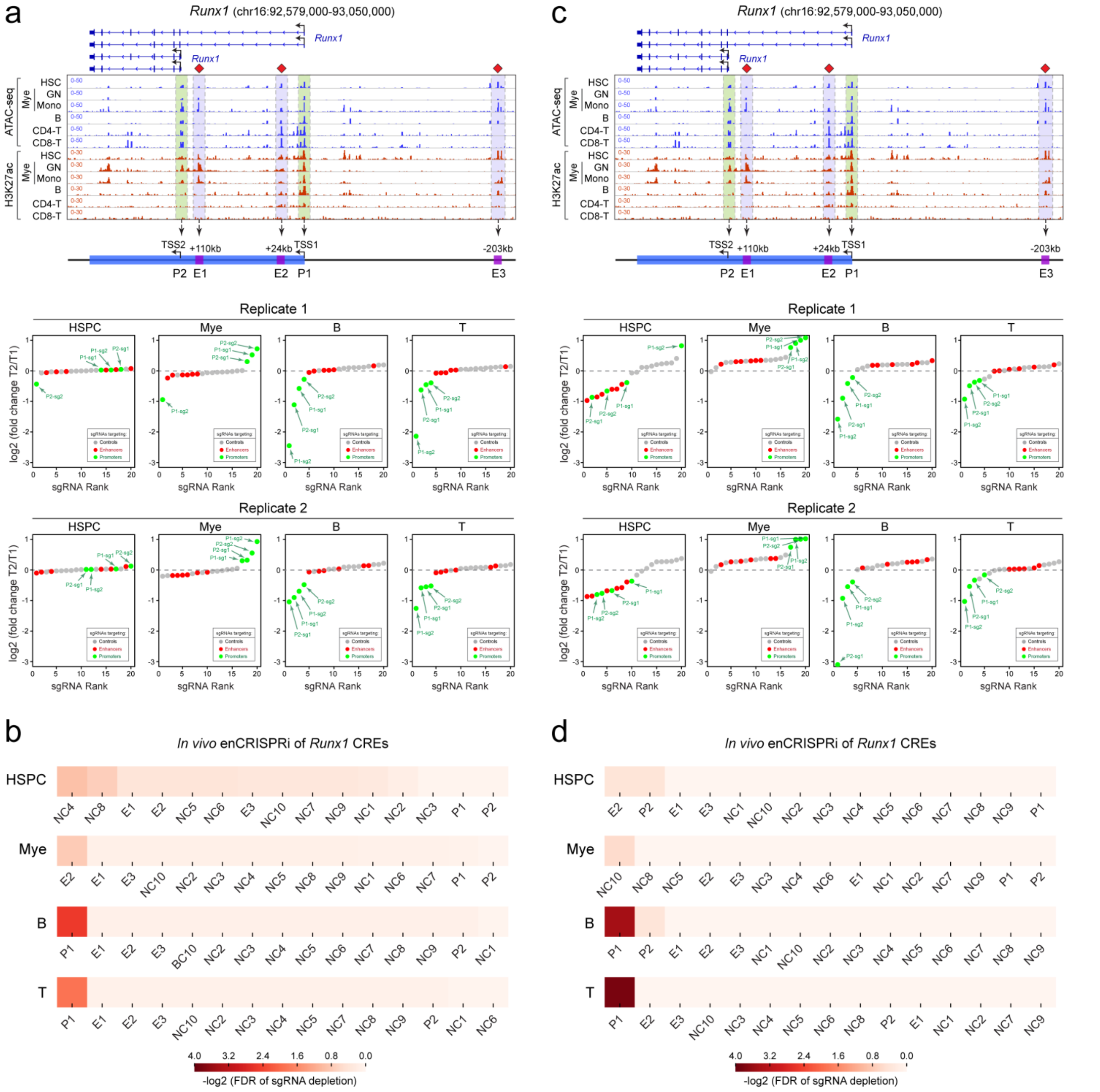

Supplementary Figure 9. Locus-Specific and Multi-Loci Perturbations of *Runx1* CREs

(a) *In vivo* enCRISPRi perturbation of *Runx1* CREs by locus-specific enCRISPRi screen during hematopoiesis. Similar to Fig. 6c, waterfall plots are shown for two independent replicate screens (*N* = 3 recipient mice per screen). The annotated *Runx1* promoters (P1 and P2) and enhancers (E1 to E3) are indicated by *green* and *blue* shaded lines.

(b) Ranking of the top depleted *Runx1* CREs by the –log2 (FDR of sgRNA depletion) by locus-specific enCRISPRi screen. The significance (FDR) of depletion for each targeted enhancer, promoter or non-targeting control (NC) region in each cell type was calculated using the MAGECK test. P, promoter; E, enhancer; NC, non-targeting control.

(c) *In vivo* enCRISPRi perturbation of *Runx1* CREs by multi-loci enCRISPRi screen during hematopoiesis. Similar to Fig. 6d, waterfall plots are shown for two independent replicate screens (*N* = 3 recipient mice per experiment).

(d) Ranking of the top depleted *Runx1* CREs by the –log2 (FDR of sgRNA depletion) in the multi-loci enCRISPRi screen.

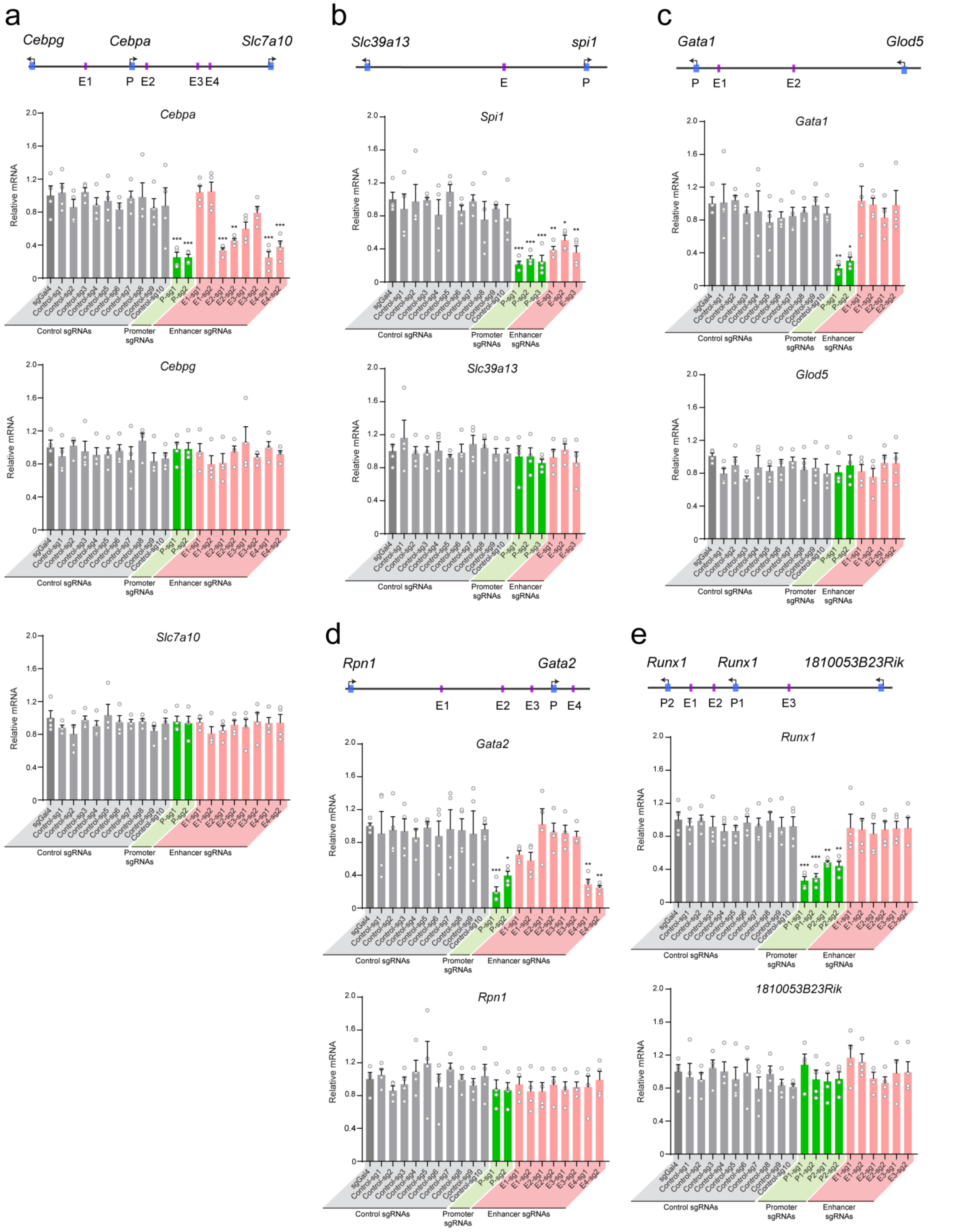

Supplementary Figure 10. *In Vivo* enCRISPRi of Promoters and Enhancers Impairs Target Gene Expression

(a) enCRISPRi-mediated perturbation of *Cebpa* CREs impaired the mRNA expression of *Cebpa* but not the other nearby genes in BM HSPCs. Independent sgRNAs (sg1 and sg2) for each targeted enhancer or promoter are shown. Cells

transduced with non-targeting sgGal4 were analyzed as the control. Results are mean ± SEM and analyzed by a one-way ANOVA. **P* < 0.05, ***P* < 0.01, ****P* < 0.001, n.s. not significant.

(b) Expression of *Spi1* and the nearby gene upon enCRISPRi-mediated perturbation of CREs in HSPCs.

(c) Expression of *Gata1* and the nearby gene upon enCRISPRi-mediated perturbation of CREs in HSPCs.

(d) Expression of *Gata2* and the nearby gene upon enCRISPRi-mediated perturbation of CREs in HSPCs.

(e) Expression of *Runx1* and the nearby gene upon enCRISPRi-mediated perturbation of CREs in HSPCs. Source data are provided as a Source Data file.

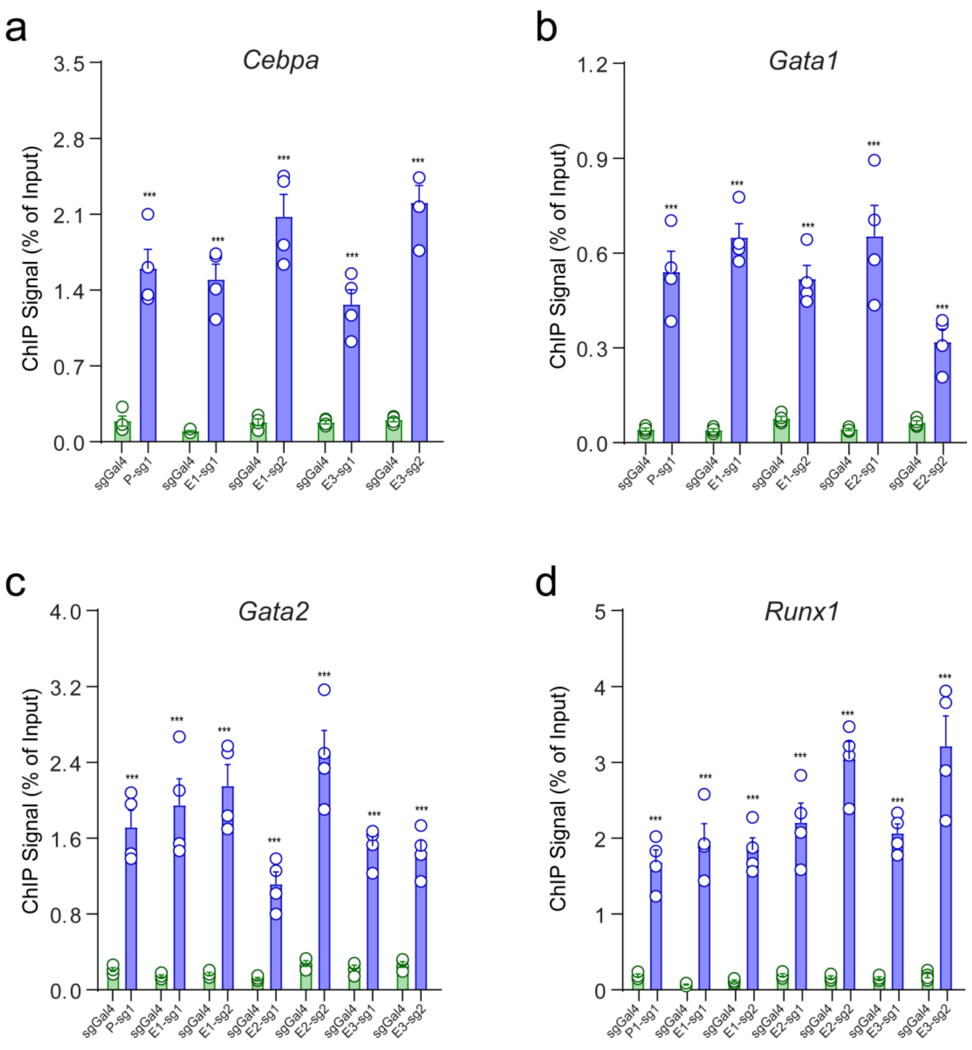

Supplementary Figure 11. Chromatin Occupancy of dCas9 in enCRISPRi-Expressing HSPCs

(a) ChIP-qPCR analysis of dCas9 occupancy in the presence of non-targeting sgRNA (sgGal4) or sgRNAs targeting *Cebpa* promoter or enhancers in BM HSPCs. Independent sgRNAs (sg1 and sg2) for each targeted enhancer or promoter are shown. Results are mean ± SEM and analyzed by a two-sided *t*-test. **P* < 0.05, ***P* < 0.01, ****P* < 0.001.

(b) ChIP-qPCR analysis of dCas9 occupancy for sgRNAs targeting *Gata1* promoter or enhancers in HSPCs.

(c) ChIP-qPCR analysis of dCas9 occupancy for sgRNAs targeting *Gata2* promoter or enhancers in HSPCs.

(d) ChIP-qPCR analysis of dCas9 occupancy for sgRNAs targeting *Runx1* promoter or enhancers in HSPCs. Source data are provided as a Source Data file.

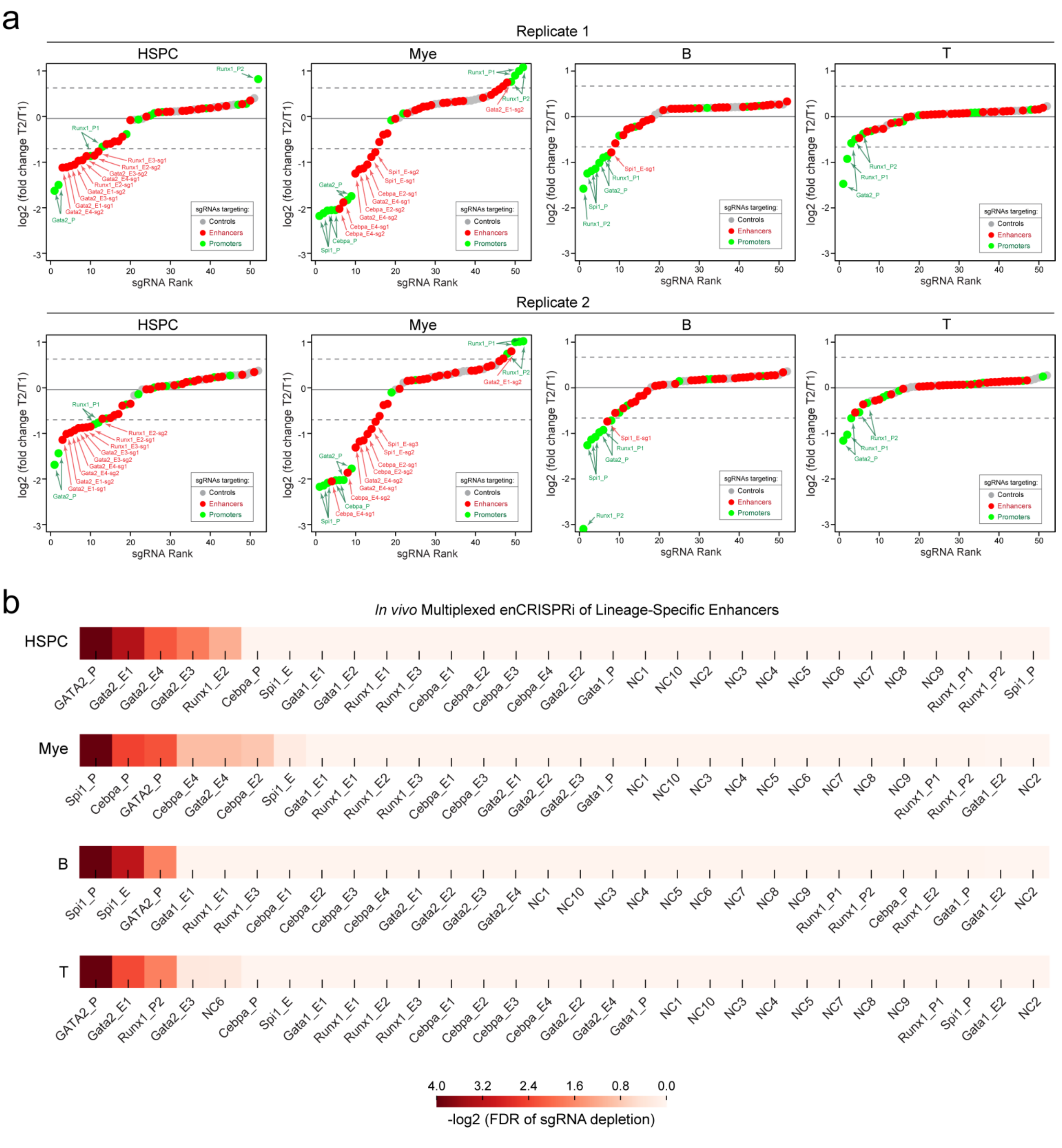

Supplementary Figure 12. Pooled sgRNA-Mediated *In Vivo* enCRISPRi Perturbations of Promoters and Enhancers during Hematopoiesis

(a) *In vivo* perturbation of annotated CREs for five key hematopoietic TFs. Similar to Fig. 6b, waterfall plots are shown for two independent replicate screens (*N* = 15 recipient mice per screen).

(b) Ranking of the top depleted CREs by the –log2 (FDR of sgRNA depletion) by multiplexed enCRISPRi screens. The significance (FDR) of depletion for each targeted enhancer, promoter or non-targeting control (NC) region in each cell type was calculated using the MAGECK test. P, promoter; E, enhancer; NC, non-targeting control.

SUPPLEMENTARY TABLES

Supplementary Table 1. List of Genomic Datasets Used in This Study

The name, data type, cell type, GEO accession number and citation for each dataset are shown.

Supplementary Table 2. Sequences of Primers and sgRNAs

The name and sequence of each primer or sgRNA are shown.

Supplementary Table 1. List of Genomic Datasets Used in This Study

Datasets	***Data Type***	***Cell Type***	***GEO ID***	***Citation***
ChIP-seq_Jurkat-H3K27ac	ChIP-seq	Jurkat	GSM3854054	This study
ChIP-seq_293T_enCRISPRa_sgGal4_HA_rep1	ChIP-seq	293T	GSM3854055	This study
ChIP-seq_293T_enCRISPRa_sgGal4_HA_rep2	ChIP-seq	293T	GSM3854056	This study
ChIP-seq_293T_enCRISPRa_sgHS2_HA_rep1	ChIP-seq	293T	GSM3854057	This study
ChIP-seq_293T_enCRISPRa_sgHS2_HA_rep2	ChIP-seq	293T	GSM3854058	This study
ChIP-seq_K562_enCRISPRi-LK_sgGal4_HA_rep1	ChIP-seq	K562	GSM3854059	This study
ChIP-seq_K562_enCRISPRi-LK_sgGal4_HA_rep2	ChIP-seq	K562	GSM3854060	This study
ChIP-seq_K562_enCRISPRi-LK_sgHS2_HA_rep1	ChIP-seq	K562	GSM3854061	This study
ChIP-seq_K562_enCRISPRi-LK_sgHS2_HA_rep2	ChIP-seq	K562	GSM3854062	This study
ChIP-seq_K562_dCas9-KRAB_sgHS2_cas9_rep1	ChIP-seq	K562	GSM3854063	This study
ChIP-seq_K562_dCas9-KRAB_sgHS2_cas9_rep2	ChIP-seq	K562	GSM3854064	This study
ChIP-seq_K562_dCas9-KRAB_sgHS2_CTCF_rep1	ChIP-seq	K562	GSM3854065	This study
ChIP-seq_K562_dCas9-KRAB_sgHS2_CTCF_rep2	ChIP-seq	K562	GSM3854066	This study
ChIP-seq_K562_dCas9-KRAB_sgHS2_GATA1_rep1	ChIP-seq	K562	GSM3854067	This study
ChIP-seq_K562_dCas9-KRAB_sgHS2_GATA1_rep2	ChIP-seq	K562	GSM3854068	This study
ChIP-seq_K562_dCas9-KRAB_sgHS2_H3K27ac_rep1	ChIP-seq	K562	GSM3854069	This study
ChIP-seq_K562_dCas9-KRAB_sgHS2_H3K27ac_rep2	ChIP-seq	K562	GSM3854070	This study
ChIP-seq_K562_dCas9-KRAB_sgHS2_H3K4me2_rep1	ChIP-seq	K562	GSM3854071	This study
ChIP-seq_K562_dCas9-KRAB_sgHS2_H3K4me2_rep2	ChIP-seq	K562	GSM3854072	This study
ChIP-seq_K562_dCas9-KRAB_sgHS2_H3K4me_rep1	ChIP-seq	K562	GSM3854073	This study
ChIP-seq_K562_dCas9-KRAB_sgHS2_H3K4me_rep2	ChIP-seq	K562	GSM3854074	This study
ChIP-seq_K562_dCas9-KRAB_sgHS2_H3K9me3_rep1	ChIP-seq	K562	GSM3854075	This study
ChIP-seq_K562_dCas9-KRAB_sgHS2_H3K9me3_rep2	ChIP-seq	K562	GSM3854076	This study
ChIP-seq_K562_dCas9-KRAB_sgHS2_TAL1_rep1	ChIP-seq	K562	GSM3854077	This study
ChIP-seq_K562_dCas9-KRAB_sgHS2_TAL1_rep2	ChIP-seq	K562	GSM3854078	This study
ChIP-seq_K562_dCas9-LSD1_sgHS2_cas9_rep1	ChIP-seq	K562	GSM3854079	This study
ChIP-seq_K562_dCas9-LSD1_sgHS2_cas9_rep2	ChIP-seq	K562	GSM3854080	This study
ChIP-seq_K562_dCas9-LSD1_sgHS2_CTCF_rep1	ChIP-seq	K562	GSM3854081	This study
ChIP-seq_K562_dCas9-LSD1_sgHS2_CTCF_rep2	ChIP-seq	K562	GSM3854082	This study
ChIP-seq_K562_dCas9-LSD1_sgHS2_GATA1_rep1	ChIP-seq	K562	GSM3854083	This study
ChIP-seq_K562_dCas9-LSD1_sgHS2_GATA1_rep2	ChIP-seq	K562	GSM3854084	This study
ChIP-seq_K562_dCas9-LSD1_sgHS2_H3K27ac_rep1	ChIP-seq	K562	GSM3854085	This study
ChIP-seq_K562_dCas9-LSD1_sgHS2_H3K27ac_rep2	ChIP-seq	K562	GSM3854086	This study
ChIP-seq_K562_dCas9-LSD1_sgHS2_H3K4me2_rep1	ChIP-seq	K562	GSM3854087	This study
ChIP-seq_K562_dCas9-LSD1_sgHS2_H3K4me2_rep2	ChIP-seq	K562	GSM3854088	This study
ChIP-seq_K562_dCas9-LSD1_sgHS2_H3K4me_rep1	ChIP-seq	K562	GSM3854089	This study
ChIP-seq_K562_dCas9-LSD1_sgHS2_H3K4me_rep2	ChIP-seq	K562	GSM3854090	This study
ChIP-seq_K562_dCas9-LSD1_sgHS2_H3K9me3_rep1	ChIP-seq	K562	GSM3854091	This study
ChIP-seq_K562_dCas9-LSD1_sgHS2_H3K9me3_rep2	ChIP-seq	K562	GSM3854092	This study
ChIP-seq_K562_dCas9-LSD1_sgHS2_TAL1_rep1	ChIP-seq	K562	GSM3854093	This study
ChIP-seq_K562_dCas9-LSD1_sgHS2_TAL1_rep2	ChIP-seq	K562	GSM3854094	This study
ChIP-seq_K562_enCRISPRi-KL_sgHS2_cas9_rep1	ChIP-seq	K562	GSM3854095	This study
ChIP-seq_K562_enCRISPRi-KL_sgHS2_cas9_rep2	ChIP-seq	K562	GSM3854096	This study
ChIP-seq_K562_enCRISPRi-KL_sgHS2_CTCF_rep1	ChIP-seq	K562	GSM3854097	This study
ChIP-seq_K562_enCRISPRi-KL_sgHS2_CTCF_rep2	ChIP-seq	K562	GSM3854098	This study
ChIP-seq_K562_enCRISPRi-KL_sgHS2_GATA1_rep1	ChIP-seq	K562	GSM3854099	This study
ChIP-seq_K562_enCRISPRi-KL_sgHS2_GATA1_rep2	ChIP-seq	K562	GSM3854100	This study
ChIP-seq_K562_enCRISPRi-KL_sgHS2_H3K27ac_rep1	ChIP-seq	K562	GSM3854101	This study
ChIP-seq_K562_enCRISPRi-KL_sgHS2_H3K27ac_rep2	ChIP-seq	K562	GSM3854102	This study
ChIP-seq_K562_enCRISPRi-KL_sgHS2_H3K4me2_rep1	ChIP-seq	K562	GSM3854103	This study
ChIP-seq_K562_enCRISPRi-KL_sgHS2_H3K4me2_rep2	ChIP-seq	K562	GSM3854104	This study
ChIP-seq_K562_enCRISPRi-KL_sgHS2_H3K4me_rep1	ChIP-seq	K562	GSM3854105	This study
ChIP-seq_K562_enCRISPRi-KL_sgHS2_H3K4me_rep2	ChIP-seq	K562	GSM3854106	This study
ChIP-seq_K562_enCRISPRi-KL_sgHS2_H3K9me3_rep1	ChIP-seq	K562	GSM3854107	This study
ChIP-seq_K562_enCRISPRi-KL_sgHS2_H3K9me3_rep2	ChIP-seq	K562	GSM3854108	This study
ChIP-seq_K562_enCRISPRi-KL_sgHS2_TAL1_rep1	ChIP-seq	K562	GSM3854109	This study
ChIP-seq_K562_enCRISPRi-KL_sgHS2_TAL1_rep2	ChIP-seq	K562	GSM3854110	This study
ChIP-seq_K562_enCRISPRi-LK_sgHS2_cas9_rep1	ChIP-seq	K562	GSM3854111	This study
ChIP-seq_K562_enCRISPRi-LK_sgHS2_cas9_rep2	ChIP-seq	K562	GSM3854112	This study
ChIP-seq_K562_enCRISPRi-LK_sgHS2_CTCF_rep1	ChIP-seq	K562	GSM3854113	This study

ChIP-seq_K562_enCRISPRi-LK_sgHS2_CTCF_rep2	ChIP-seq	K562	GSM3854114	This study
ChIP-seq_K562_enCRISPRi-LK_sgHS2_GATA1_rep1	ChIP-seq	K562	GSM3854115	This study
ChIP-seq_K562_enCRISPRi-LK_sgHS2_GATA1_rep2	ChIP-seq	K562	GSM3854116	This study
ChIP-seq_K562_enCRISPRi-LK_sgHS2_H3K27ac_rep1	ChIP-seq	K562	GSM3854117	This study
ChIP-seq_K562_enCRISPRi-LK_sgHS2_H3K27ac_rep2	ChIP-seq	K562	GSM3854118	This study
ChIP-seq_K562_enCRISPRi-LK_sgHS2_H3K4me2_rep1	ChIP-seq	K562	GSM3854119	This study
ChIP-seq_K562_enCRISPRi-LK_sgHS2_H3K4me2_rep2	ChIP-seq	K562	GSM3854120	This study
ChIP-seq_K562_enCRISPRi-LK_sgHS2_H3K4me_rep1	ChIP-seq	K562	GSM3854121	This study
ChIP-seq_K562_enCRISPRi-LK_sgHS2_H3K4me_rep2	ChIP-seq	K562	GSM3854122	This study
ChIP-seq_K562_enCRISPRi-LK_sgHS2_H3K9me3_rep1	ChIP-seq	K562	GSM3854123	This study
ChIP-seq_K562_enCRISPRi-LK_sgHS2_H3K9me3_rep2	ChIP-seq	K562	GSM3854124	This study
ChIP-seq_K562_enCRISPRi-LK_sgHS2_TAL1_rep1	ChIP-seq	K562	GSM3854125	This study
ChIP-seq_K562_enCRISPRi-LK_sgHS2_TAL1_rep2	ChIP-seq	K562	GSM3854126	This study
ChIP-seq_K562_sgGal4_Cas9_rep1	ChIP-seq	K562	GSM3854127	This study
ChIP-seq_K562_sgGal4_Cas9_rep2	ChIP-seq	K562	GSM3854128	This study
ChIP-seq_K562_sgGal4_CTCF_rep1	ChIP-seq	K562	GSM3854129	This study
ChIP-seq_K562_sgGal4_CTCF_rep2	ChIP-seq	K562	GSM3854130	This study
ChIP-seq_K562_sgGal4_GATA1_rep1	ChIP-seq	K562	GSM3854131	This study
ChIP-seq_K562_sgGal4_GATA1_rep2	ChIP-seq	K562	GSM3854132	This study
ChIP-seq_K562_sgGal4_H3K27ac_rep1	ChIP-seq	K562	GSM3854133	This study
ChIP-seq_K562_sgGal4_H3K27ac_rep2	ChIP-seq	K562	GSM3854134	This study
ChIP-seq_K562_sgGal4_H3K4me2_rep1	ChIP-seq	K562	GSM3854135	This study
ChIP-seq_K562_sgGal4_H3K4me2_rep2	ChIP-seq	K562	GSM3854136	This study
ChIP-seq_K562_sgGal4_H3K4me_rep1	ChIP-seq	K562	GSM3854137	This study
ChIP-seq_K562_sgGal4_H3K4me_rep2	ChIP-seq	K562	GSM3854138	This study
ChIP-seq_K562_sgGal4_H3K9me3_rep1	ChIP-seq	K562	GSM3854139	This study
ChIP-seq_K562_sgGal4_H3K9me3_rep2	ChIP-seq	K562	GSM3854140	This study
ChIP-seq_K562_sgGal4_TAL1_rep1	ChIP-seq	K562	GSM3854141	This study
ChIP-seq_K562_sgGal4_TAL1_rep2	ChIP-seq	K562	GSM3854142	This study
ChIP-seq_K562_dCas9-KRAB_sgHS2_H3K27me3_rep1	ChIP-seq	K562	GSM4190316	This study
ChIP-seq_K562_dCas9-KRAB_sgHS2_H3K27me3_rep2	ChIP-seq	K562	GSM4190317	This study
ChIP-seq_K562_dCas9-LSD1_sgHS2_H3K27me3_rep1	ChIP-seq	K562	GSM4190318	This study
ChIP-seq_K562_dCas9-LSD1_sgHS2_H3K27me3_rep2	ChIP-seq	K562	GSM4190319	This study
ChIP-seq_K562_enCRISPRi-KL_sgHS2_H3K27me3_rep1	ChIP-seq	K562	GSM4190320	This study
ChIP-seq_K562_enCRISPRi-KL_sgHS2_H3K27me3_rep2	ChIP-seq	K562	GSM4190321	This study
ChIP-seq_K562_enCRISPRi-LK_sgHS2_H3K27me3_rep1	ChIP-seq	K562	GSM4190322	This study
ChIP-seq_K562_enCRISPRi-LK_sgHS2_H3K27me3_rep2	ChIP-seq	K562	GSM4190323	This study
ChIP-seq_K562_sgGal4_H3K27me3_rep1	ChIP-seq	K562	GSM4190324	This study
ChIP-seq_K562_sgGal4_H3K27me3_rep2	ChIP-seq	K562	GSM4190325	This study
RNA-seq_K562_dCas9-KRAB_sgHS2_rep1	RNA-seq	K562	GSM3854143	This study
RNA-seq_K562_dCas9-KRAB_sgHS2_rep2	RNA-seq	K562	GSM3854144	This study
RNA-seq_K562_dCas9-LSD1_sgHS2_rep1	RNA-seq	K562	GSM3854145	This study
RNA-seq_K562_dCas9-LSD1_sgHS2_rep2	RNA-seq	K562	GSM3854146	This study
RNA-seq_K562_enCRISPRi-KL_sgHS2_rep1	RNA-seq	K562	GSM3854147	This study
RNA-seq_K562_enCRISPRi-KL_sgHS2_rep2	RNA-seq	K562	GSM3854148	This study
RNA-seq_K562_enCRISPRi-LK_sgHS2_rep1	RNA-seq	K562	GSM3854149	This study
RNA-seq_K562_enCRISPRi-LK_sgHS2_rep2	RNA-seq	K562	GSM3854150	This study
RNA-seq_K562_sgGal4_rep1	RNA-seq	K562	GSM3854151	This study
RNA-seq_K562_sgGal4_rep2	RNA-seq	K562	GSM3854152	This study
ATAC-seq_Jurkat	ATAC-seq	Jurkat	GSM3854041	This study
ATAC_seq_B	ATAC-seq	B cell	GSM1463172	Lara-Astiaso et al, Science 2014
ATAC_seq_Monocytes	ATAC-seq	Monocytes	GSM1463174	Lara-Astiaso et al, Science 2014
ATAC_seq_CD4	ATAC-seq	T cell	GSM1463175	Lara-Astiaso et al, Science 2014
ATAC_seq_Granulocytes	ATAC-seq	Granulocytes	GSM1463176	Lara-Astiaso et al, Science 2014
ATAC_seq_CD8	ATAC-seq	T cell	GSM1463178	Lara-Astiaso et al, Science 2014
ATAC_seq_LSK	ATAC-seq	LSK cell	GSM1463179	Lara-Astiaso et al, Science 2014
ChIP-seq_H3K27Ac_LT_HSC	ChIP-seq	LT-HSC	GSM1441269	Lara-Astiaso et al, Science 2014
ChIP-seq_H3K27Ac_GN	ChIP-seq	Granulocytes	GSM1441277	Lara-Astiaso et al, Science 2014
ChIP-seq_H3K27Ac_Mono	ChIP-seq	Monocytes	GSM1441278	Lara-Astiaso et al, Science 2014
ChIP-seq_H3K27Ac_B	ChIP-seq	B cell	GSM1441280	Lara-Astiaso et al, Science 2014
ChIP-seq_H3K27Ac_CD4	ChIP-seq	T cell	GSM1441281	Lara-Astiaso et al, Science 2014
ChIP-seq_H3K27Ac_CD8	ChIP-seq	T cell	GSM1441282	Lara-Astiaso et al, Science 2014

Supplementary Table 2. Sequences of Primers and sgRNAs

Name	Forward	Reverse	Application
HS2-1-sgRNA	CACCGAATATGTCACATTCTGTCTC	AAACGAGACAGAATGTGACATATTC	sgRNA for enhancer or promoter perturbation
HS2-2-sgRNA	CACCGGGACTATGGGAGGTCACTAA	AAACTTAGTGACCTCCCATAGTCCC	
HS2-3-sgRNA	CACCGGAAGGTTACACAGAACCAGA	AAACTCTGGTTCTGTGTAACCTTCC	
HS2-4-sgRNA	CACCGGCCCTGTAAGCATCCTGCTG	AAACCAGCAGGATGCTTACAGGGCC	
IL1RN-sgRNA1	CACCGTGTACTCTCTGAGGTGCTC	AAACGAGCACCTCAGAGAGTACAC	
IL1RN-sgRNA2	CACCGCATCAAGTCAGCCATCAGC	AAACGCTGATGGCTGACTTGATGC	
OCT4-sgRNA1	CACCGACTCCACTGCACTCCAGTCT	AAACAGACTGGAGTGCAGTGGAGTC	
OCT4-sgRNA2	CACCGACACCATTGCCACCACCATT	AAACAATGGTGGTGGCAATGGTGTC	
MYOD-sgRNA1	CACCGGGCCCACATTCCTTTCCAG	AAACCTGGAAAGGAATGTGGGCCC	
MYOD-sgRNA2	CACCGGGCTGGATTGGGTTTCCAG	AAACCTGGAAACCCAATCCAGCCC	
Gal4_sgRNA	CACCGAACGACTAGTTAGGCGTGTA	AAACTACACGCCTAACTAGTCGTTC	
Ctrl-sgRNA	CACCGTAGCGGGTAAGGATGTAGAC	AAACGTCTACATCCTTACCCGCTAC	
TAD1-sgRNA	CACCGTTGAACTAGTCCCGATAGGA	AAACTCCTATCGGGACTAGTTCAAC	
CTCF1-sgRNA	CACCGACCAGAAACACATCCCAGAT	AAACATCTGGGATGTGTTTCTGGTC	
CTCF2-sgRNA	CACCGTTAGACAGCAATGTTTATCC	AAACGGATAAACATTGCTGTCTAAC	
TAD2-sgRNA	CACCGCAGCGAGACTCCGTCGGCGT	AAACACGCCGACGGAGTCTCGCTGC	
HS1-sgRNA	CACCGCAATAGGTATATGAGGAGAC	AAACGTCTCCTCATATACCTATTGC	
HS2-sgRNA	CACCGGCCCTGTAAGCATCCTGCTG	AAACCAGCAGGATGCTTACAGGGCC	
HS3-sgRNA	CACCGTTTGGGAGCAGGAGTCTCTA	AAACTAGAGACTCCTGCTCCCAAAC	
HS4-sgRNA	CACCGCCCACTCAGCAGCTATGAGA	AAACTCTCATAGCTGCTGAGTGGGC	
HS5-sgRNA	CACCGTGCCCCCACCTTACAGGGAC	AAACGTCCCTGTAAGGTGGGGGCAC	
HS2+2.5K-sgRNA	CACCGCACAGTCTCTACCTCAGAAT	AAACATTCTGAGGTAGAGACTGTGC	
HS2+0.5K-sgRNA	CACCGGGCACCCCTGGACAGCTAGG	AAACCCTAGCTGTCCAGGGGTGCCC	
HS2-0k-sgRNA	CACCGGAAGGTTACACAGAACCAGA	AAACTCTGGTTCTGTGTAACCTTCC	
HS2-2.5K-sgRNA	CACCGATCCTAGCCTTACTACTTGG	AAACCCAAGTAGTAAGGCTAGGATC	
HS2-0.5K-sgRNA	CACCGAATAGAGGTATTTATGGGAT	AAACATCCCATAAATACCTCTATTC	
HBG-sgRNA	CACCGGCTAAACTCCACCCATGGGT	AAACACCCATGGGTGGAGTTTAGCC	
TAL1-Mut-sgRNA1	CACCGagggtCACAGAAAGACGGTT	AAACAACCGTCTTTCTGTGaccctC	
TAL1-Mut-sgRNA2	CACCGACAGAAAGACGGTTAGGAAA	AAACTTTCCTAACCGTCTTTCTGTC	
TAL1-WT-sgRNA1	CACCGGAATGGGGTGGGGCAACCAC	AAACGTGGTTGCCCCACCCCATTCC	
TAL1-WT-sgRNA2	CACCGAAAGACGTAACCCTACTTCC	AAACGGAAGTAGGGTTACGTCTTTC	
Spi1-P-sg1	caccgCAAGTTCCTGATTTTATCGA	aaacTCGATAAAATCAGGAACTTGc	
Spi1-P-sg2	caccgGCCCCCTCCCTTGACATTGC	aaacGCAATGTCAAGGGAGGGGGCc	
Spi1-P-sg3	caccgACGGTCGTGGGTCAGACGCA	aaacTGCGTCTGACCCACGACCGTc	
Spi1-Enh-sg1	caccgAGGAAGCGCCCAGTCACCAC	aaacGTGGTGACTGGGCGCTTCCTc	
Spi1-Enh-sg2	caccgTGCCCAGCCTGGAGCCGCCC	aaacGGGCGGCTCCAGGCTGGGCAc	
Spi1-Enh-sg3	caccgAGCTGCCCCTGTTTCCACAT	aaacATGTGGAAACAGGGGCAGCTc	
Cebpa-P-sg1	caccgGTGCTAGTGGAGAGAGATCG	aaacCGATCTCTCTCCACTAGCACc	
Cebpa-P-sg2	caccgACTTTCCAAGGCGGTGAGTG	aaacCACTCACCGCCTTGGAAAGTc	
Cebpa-Enh1-sg1	caccgCTCCGTTCTTTTAATAGCGC	aaacGCGCTATTAAAAGAACGGAGc	
Cebpa-Enh1-sg2	caccgTTGCTGCTGCTTTCTACCAC	aaacGTGGTAGAAAGCAGCAGCAAc	
Cebpa-Enh2-sg1	caccgGTAATTGCCCCGGCAGAACT	aaacAGTTCTGCCGGGGCAATTACc	
Cebpa-Enh2-sg2	caccgTCTGACGGGTCCTAACCTGA	aaacTCAGGTTAGGACCCGTCAGAc	
Cebpa-Enh3-sg1	caccgCTTCTCATCACGGATCAAAA	aaacTTTTGATCCGTGATGAGAAGc	
Cebpa-Enh3-sg2	caccgTGTTTCTGTTTTGGGGCCAC	aaacGTGGCCCCAAAACAGAAACAc	
Cebpa-Enh4-sg1	caccgAAACGGGAAGTACTGAGCTA	aaacTAGCTCAGTACTTCCCGTTTc	
Cebpa-Enh4-sg2	caccgGCGGGGTGAGGACATATCTC	aaacGAGATATGTCCTCACCCCGCc	
Gata2-P-sg1	caccgGTCTCGGCCTCCGGCCCGCC	aaacGGCGGGCCGGAGGCCGAGACc	
Gata2-P-sg2	caccgCCTGCACAGACGTGAAGCGG	aaacCCGCTTCACGTCTGTGCAGGc	
Gata2-Enh1-sg1	caccgAGGTGGCCCACGGGGTGGCG	aaacCGCCACCCCGTGGGCCACCTc	
Gata2-Enh1-sg2	caccgCCCTGCAGCAGGGGTTCTGC	aaacGCAGAACCCCTGCTGCAGGGc	
Gata2-Enh2-sg1	caccgAACTTACGGAAACCACCTTG	aaacCAAGGTGGTTTCCGTAAGTTc	
Gata2-Enh2-sg2	caccgAGGTGGATGGCCGGGCCACT	aaacAGTGGCCCGGCCATCCACCTc	
Gata2-Enh3-sg1	caccgATGGCGACGCAGATAGCGCG	aaacCGCGCTATCTGCGTCGCCATc	
Gata2-Enh3-sg2	caccgCCCGGAGTCTATTATACAGC	aaacGCTGTATAATAGACTCCGGGc	
Gata2-Enh4-sg1	caccgACTCCGGCAGGAGATCCGAA	aaacTTCGGATCTCCTGCCGGAGTc	
Gata2-Enh4-sg2	caccgGCTCTGAAAACTTGCCGGTC	aaacGACCGGCAAGTTTTCAGAGCc	
Gata1-P-sg1	caccgCGGAGGGACTAGAGCCTAAA	aaacTTTAGGCTCTAGTCCCTCCGc	
Gata1-P-sg2	caccgAAGGGGATCCAACAACCTGC	aaacGCAGGTTGTTGGATCCCCTTc	
Gata1-Enh1-sg1	caccgATAGATAAGGGAATCAGCGG	aaacCCGCTGATTCCCTTATCTATc	
Gata1-Enh1-sg2	caccgACAGAGAAGACGCTTCAACC	aaacGGTTGAAGCGTCTTCTCTGTc	
Gata1-Enh2-sg1	caccgACTATTTGTGCGGAAGTCTC	aaacGAGACTTCCGCACAAATAGTc	
Gata1-Enh2-sg2	caccgGTAAAGTGACAGTAAAGCGA	aaacTCGCTTTACTGTCACTTTACc	
Runx1-P1-sg1	caccgGGAATGGAATTCCTCGGGTC	aaacGACCCGAGGAATTCCATTCCc	
Runx1-P1-sg2	caccgGTAAAAGCACGTTCATTTCC	aaacGGAAATGAACGTGCTTTTACc	
Runx1-P2-sg1	caccgGAACCACAAGTTGGGTAGCC	aaacGGCTACCCAACTTGTGGTTCc	
Runx1-P2-sg2	caccgGGGTTGTGTGAAAACTTCTT	aaacAAGAAGTTTTCACACAACCCc	
Runx1-Enh1-sg1	caccgGCATTATCTGCCTTCACAAG	aaacCTTGTGAAGGCAGATAATGCc	
Runx1-Enh1-sg2	caccgATCCCCGCCCCAGCGACAGT	aaacACTGTCGCTGGGGCGGGGATc	
Runx1-Enh2-sg1	caccgTGCAAGAGCGAGAAAACCGC	aaacGCGGTTTTCTCGCTCTTGCAc	
Runx1-Enh2-sg2	caccgATGCTGACAGCCTCAGATGG	aaacCCATCTGAGGCTGTCAGCATc	
Runx1-Enh3-sg1	caccgTGTGCGAGCATTGCCCCAGC	aaacGCTGGGGCAATGCTCGCACAc	
Runx1-Enh3-sg2	caccgGATCTGAGTTTAGTCACTTT	aaacAAAGTGACTAAACTCAGATCc	
NC-sg1	caccgGAGGTTGCAAGGGACCCGGT	aaacACCGGGTCCCTTGCAACCTCc	
NC-sg2	caccgGCATTTGAGGCACACTGGAG	aaacCTCCAGTGTGCCTCAAATGCc	
NC-sg3	caccgGCAGTTGCACAGATGATCAC	aaacGTGATCATCTGTGCAACTGCc	
NC-sg4	caccgGGTGGTGTCGTAGTGTCAGA	aaacTCTGACACTACGACACCACCc	
NC-sg5	caccgGCCTATGCTACGGACTATAG	aaacCTATAGTCCGTAGCATAGGCc	
NC-sg6	caccgGGTGGCCTGGTACAGCGCAG	aaacCTGCGCTGTACCAGGCCACCc	
NC-sg7	caccgGGGGCCTGACCACGATAGCA	aaacTGCTATCGTGGTCAGGCCCCc	
NC-sg8	caccgGAGAGGGTTCAAAGCAGGCG	aaacCGCCTGCTTTGAACCCTCTCc	
NC-sg9	caccgGCGCGAAAGGCGAGATTAAC	aaacGTTAATCTCGCCTTTCGCGCc	
NC-sg10	caccgGCAGCCAAACGAAAAGACCG	aaacCGGTCTTTTCGTTTGGCTGCc	
IL1RN-RT	GGAATCCATGGAGGGAAGAT	TGTTCTCGCTCAGGTCAGTG	RT-qPCR primers
MYOD-RT	TCCCTCTTTCACGGTCTCAC	AACACCCGACTGCTGTATCC	
OCT4-RT	CGAAAGAGAAAGCGAACCAGTATCGAGAAC	CGTTGTGCATAGTCGCTGCTTGATCGC	
HBE1-RT	GCAAGAAGGTGCTGACTTCC	ACCATCACGTTACCCAGGAG	
HBG-RT	TGGATGATCTCAAGGGCAC	TCAGTGGTATCTGGAGGACA	
HBB-RT	CTGAGGAGAAGTCTGCCGTTA	AGCATCAGGAGTGGACAGAT	
GAPDH-RT	ACCCAGAAGACTGTGGATGG	TTCAGCTCAGGGATGACCTT	
TAL1-RT	GTGCAGCTGAGTCCTCCCGC	CCGGCTCCCCAAAGAACCCG	
Spi1-RT	TACCAACGTCCAATGCATGACTAC	GAGTATCGAGGACGTGCATCTGTT	
Runx1-RT	CCAGCAAGCTGAGGAGCGGCG	TGACGGTGACCAGAGTG	
Gata1-RT	CAGAACCGGCCTCTCATCC	TAGTGCATTGGGTGCCTGC	
Gata2-RT	GCAGAGAAGCAAGGCTCGC	CAGTTGACACACTCCCGGC	
Cebpa-1-RT	GCGGGAACGCAACAACATC	GTCACTGGTCAACTCCAGCAC	
EP300-RT	AGCCAAGCGGCCTAAACTC	TCACCACCATTGGTTAGTCCC	
ANKRD20A2-RT	CAATATAAGAAAGAAGTTGAAGC	TCCATCAGACCTTTCATTTCTTC	
ANKRD30BL-RT	TGCTGTTGCTTGTGGATTTAATCTC	TTCAGCCGTGTCAGGTGTTCTTTC	
ZNF806-RT	GATAAACCTGTACCAAGACGTG	TCCTTTGTTTCCAAATCTTCCAG	
LOC101928195-RT	AAGATAAGGACCATTGCCCAGG	AGGTAAGATGAAGTCCCTTGGCAC	
Cebpg_RT	AGCGGCTTACAGCAGGTTC	GGCGGTATTCGTCACTATTCC	
Slc7a10_RT	GGGTTTGGCCCTCTTCGTC	GACATAGGCGTAGTCCCCAC	
Slc39a13_RT	AGTGGCTATCTCAACCTGCTT	GCCCGATCTTTTTGCTCACAA	
Glod5_RT	CCCTTCCCCGTGTCTTATCTG	GCCCAGGATCTTGGAATAAAACA	
Rpn1_RT	GCTCCACATCACGAGCCAG	CAGTTTCCACAACGACCGAGA	
1810053B23Rik_RT	CTTCACTCTTGGGATGACCTTGCTC	CCTTATGTTTGAGAGGTGGCATC	
Cebpa-P-ChIP	TTGCGCCACGATCTCTCTCCAC	TTTATAGAGGGTCGGGCATCGCC	ChIP primers
Cebpa-Enh1-ChIP	CCAACTGTGCTTTGC TGCTGCTTTC	GTATAGAGAGGGAAACAGATGACC	
Cebpa-Enh3-ChIP	CCGTGATGAGAAGAGGAAGTCCTG	GATAGCATCGGGATTTGGTGTAG	
Gata2-P-ChIP	CGGGCTCTCCTGGTGTCTCTTACTC	CCACAGAGATCCCTCCAACCTAG	
Gata2-Enh1-ChIP	TGTCTCACCATCCCTGGGCCTTC	GATAGATAGTTTGTGAAACACC	
Gata2-Enh2-ChIP	GCCGACTACCACTTTAGGCTTGC	TCCGGCATGGCAGTTGGTGAAG	
Gata2-Enh3-ChIP	CGCCGCAGGTTGTGTGACTTTGG	ATGGTCCCCATGGCGACGCAGA	
Gata1-P-ChIP	CCCT TTGCCTTTGGCTTCTGTGGAG	GCATACAGGTCCAACCCACACATAG	
Gata1-Enh1-ChIP	AAGGGCTCAGTAAAGGAAATGAAGG	CTGATTCCCTTATCTATGCCTTC	
Gata1-Enh2-ChIP	ACTGGAGTTAAGGATGGTTGGGAGC	CCTAACTACTGTGGGCCACCTC	
Runx1-P1-ChIP	AAAGCCTGTGGTTTGCATTCAGTG	CAATGGCGGTGGGCTGTGGAAAG	
Runx1-Enh1-ChIP	TTCCTCAGGAGAAGTGTCTTAG	CAAAGCCCAGGAAACACCTTGCAG	
Runx1-Enh2-ChIP	CGATGGAGGGATGGTGTGAGGAG	GATGGAGGCATCCTGTTTGTCG	
Runx1-Enh3-ChIP	AGAGCAGAAGGAACTCAGAAGGCAG	AAATGGTCAGTCTGCTGAAGATC	
rtTA-A	AAAGTCGCTCTGAGTTGTTAT		Mouse genotyping primers
rtTA-B	GCGAAGAGTTTGTCCTCAACC		
rtTA-C	GGAGCGGGAGAAATGGATATG		
dCas9-KRAB-A	GCACAGCATTGCGGACATGC		
dCas9-KRAB-B	CCCTCCATGTGTGACCAAGG		
dCas9-KRAB-C	GCAGAAGCGCGGCCGTCTGG		
TAL1_WT_ChIP	GAAGCAGAGTGAAAGAGATGATAAG	TCTGCCAGGAAGTAGGGTTACGTC	TAL1 super-enhancer ChIP primers
TAL1_Mut_ChIP	GAAGCAGAGTGAAAGAGATGATAAG	AGTAGGGTTACCGTTTCCTAAC	
gDNA_sgRNA_NGS_LCreadoutPRC1-F	AATGGACTATCATATGCTTACCGTAACTTGAAAGTATTTCG	AAAAAAAgcaccgactcggtgccacttggccCTGCAGACA	

sgRNApool_NGS_F1	AATGATACGGCGACCACCGAGATCTACACTCTTTCCCTACACGACGCTCTTCCGATCTtAAGTAGAGtcttgtggaaaggacgaaacaccg		sgRNA pooled screen primers with barcodes and staggers
sgRNApool_NGS_F2	AATGATACGGCGACCACCGAGATCTACACTCTTTCCCTACACGACGCTCTTCCGATCTatACACGATCtcttgtggaaaggacgaaacaccg		
sgRNApool_NGS_F3	AATGATACGGCGACCACCGAGATCTACACTCTTTCCCTACACGACGCTCTTCCGATCTgatCGCGCGGTtcttgtggaaaggacgaaacaccg		
sgRNApool_NGS_F4	AATGATACGGCGACCACCGAGATCTACACTCTTTCCCTACACGACGCTCTTCCGATCTcgatCATGATCGtcttgtggaaaggacgaaacaccg		
sgRNApool_NGS_F5	AATGATACGGCGACCACCGAGATCTACACTCTTTCCCTACACGACGCTCTTCCGATCTtcgatCGTTACCAtcttgtggaaaggacgaaacaccg		
sgRNApool_NGS_F6	AATGATACGGCGACCACCGAGATCTACACTCTTTCCCTACACGACGCTCTTCCGATCTatcgatTCCTTGGTtcttgtggaaaggacgaaacaccg		
sgRNApool_NGS_F7	AATGATACGGCGACCACCGAGATCTACACTCTTTCCCTACACGACGCTCTTCCGATCTgatcgatAACGCATTtcttgtggaaaggacgaaacaccg		
sgRNApool_NGS_F8	AATGATACGGCGACCACCGAGATCTACACTCTTTCCCTACACGACGCTCTTCCGATCTcgatcgatACAGGTATtcttgtggaaaggacgaaacaccg		
sgRNApool_NGS_F9	AATGATACGGCGACCACCGAGATCTACACTCTTTCCCTACACGACGCTCTTCCGATCTacgatcgatAGGTAAGGtcttgtggaaaggacgaaacaccg		
sgRNApool_NGS_F10	AATGATACGGCGACCACCGAGATCTACACTCTTTCCCTACACGACGCTCTTCCGATCTtAACAATGGtcttgtggaaaggacgaaacaccg		
sgRNApool_NGS_F11	AATGATACGGCGACCACCGAGATCTACACTCTTTCCCTACACGACGCTCTTCCGATCTatACTGTATCtcttgtggaaaggacgaaacaccg		
sgRNApool_NGS_F12	AATGATACGGCGACCACCGAGATCTACACTCTTTCCCTACACGACGCTCTTCCGATCTgatAGGTCGCAtcttgtggaaaggacgaaacaccg		
PMLS_POOL_NGS_R1	CAAGCAGAAGACGGCATACGAGATAAGTAGAGGTGACTGGAGTTCAGACGTGTGCTCTTCCGATCTtTCGGTGCCACTTGGCCCTGCAGACA		
PMLS_POOL_NGS_R2	CAAGCAGAAGACGGCATACGAGATACACGATCGTGACTGGAGTTCAGACGTGTGCTCTTCCGATCTatTCGGTGCCACTTGGCCCTGCAGACA		
PMLS_POOL_NGS_R3	CAAGCAGAAGACGGCATACGAGATCGCGCGGTGTGACTGGAGTTCAGACGTGTGCTCTTCCGATCTgatTCGGTGCCACTTGGCCCTGCAGACA		
PMLS_POOL_NGS_R4	CAAGCAGAAGACGGCATACGAGATCATGATCGGTGACTGGAGTTCAGACGTGTGCTCTTCCGATCTcgatTCGGTGCCACTTGGCCCTGCAGACA		
PMLS_POOL_NGS_R5	CAAGCAGAAGACGGCATACGAGATCGTTACCAGTGACTGGAGTTCAGACGTGTGCTCTTCCGATCTcgatcTCGGTGCCACTTGGCCCTGCAGACA		
PMLS_POOL_NGS_R6	CAAGCAGAAGACGGCATACGAGATTCCTTGGTGTGACTGGAGTTCAGACGTGTGCTCTTCCGATCTtTCGGTGCCACTTGGCCCTGCAGACA		
