# Supplementary material for: Interrogation of enhancer function by enhancer-targeting CRISPR epigenetic editing
Source: Nat Commun. 2020 Jan 24;11:485. doi: 10.1038/s41467-020-14362-5 (PMC6981169; doi:10.1038/s41467-020-14362-5)
Supplement: Supplementary file 3 — Reporting Summary [file 41467_2020_14362_MOESM3_ESM.pdf]

nature research

Corresponding author(s): Jian Xu

Last updated by author(s): Dec 31, 2019

Reporting Summary

Nature Research wishes to improve the reproducibility of the work that we publish. This form provides structure for consistency and transparency in reporting. For further information on Nature Research policies, see Authors & Referees and the Editorial Policy Checklist .

Statistics

For all statistical analyses, confirm that the following items are present in the figure legend, table legend, main text, or Methods section.

n/a	Confirmed	
☐	☒	The exact sample size (*n*) for each experimental group/condition, given as a discrete number and unit of measurement
☐	☒	A statement on whether measurements were taken from distinct samples or whether the same sample was measured repeatedly
☐	☒	The statistical test(s) used AND whether they are one- or two-sided<br>*Only common tests should be described solely by name; describe more complex techniques in the Methods section.*
☒	☐	A description of all covariates tested
☐	☒	A description of any assumptions or corrections, such as tests of normality and adjustment for multiple comparisons
☐	☒	A full description of the statistical parameters including central tendency (e.g. means) or other basic estimates (e.g. regression coefficient) AND variation (e.g. standard deviation) or associated estimates of uncertainty (e.g. confidence intervals)
☒	☐	For null hypothesis testing, the test statistic (e.g. *F*, *t*, *r*) with confidence intervals, effect sizes, degrees of freedom and *P* value noted<br>*Give P values as exact values whenever suitable.*
☒	☐	For Bayesian analysis, information on the choice of priors and Markov chain Monte Carlo settings
☒	☐	For hierarchical and complex designs, identification of the appropriate level for tests and full reporting of outcomes
☒	☐	Estimates of effect sizes (e.g. Cohen's *d*, Pearson's *r*), indicating how they were calculated

Our web collection on statistics for biologists contains articles on many of the points above.

Software and code

Policy information about availability of computer code

Data collection	No software was used.
Data analysis	MACS version 2: for ChIP-seq peak calling<br>Bowtie version 2.1.0: for alignment<br>STAR 2.5.2b: for RNA-seq read alighment<br>RSEM: for calculation of normalized gene expression<br>DESeq2: for differential gene expression analysis<br>MAnorm: for comparison of ChIP-seq signal intensities in different samples

For manuscripts utilizing custom algorithms or software that are central to the research but not yet described in published literature, software must be made available to editors/reviewers. We strongly encourage code deposition in a community repository (e.g. GitHub). See the Nature Research guidelines for submitting code & software for further information.

Data

Policy information about availability of data

All manuscripts must include a data availability statement. This statement should provide the following information, where applicable:
- Accession codes, unique identifiers, or web links for publicly available datasets
- A list of figures that have associated raw data
- A description of any restrictions on data availability

All raw and processed ATAC-seq, ChIP-seq and RNA-seq data are available in the Gene Expression Omnibus (GEO): GSE132216

Field-specific reporting

Please select the one below that is the best fit for your research. If you are not sure, read the appropriate sections before making your selection.

- [x] Life sciences
- [ ] Behavioural & social sciences
- [ ] Ecological, evolutionary & environmental sciences

For a reference copy of the document with all sections, see nature.com/documents/nr-reporting-summary-flat.pdf

Life sciences study design

All studies must disclose on these points even when the disclosure is negative.

Sample size	A sample size of 3 (for locus-specific perturbations) or 15 (for multi-loci perturbations) recipient mice was selected for in vivo enCRISPRi perturbation screens. A sample size of two independent replicate screens were performed. A sample size of two independent replicates was performed for ChIP-seq and RNA-seq experiments following enCRISPRi or enCRISPRa. These sample sizes were selected because multiple statistical approaches have been developed to allow identification of significantly changed genes or genomic loci from two or more biological replicates of high-throughput sequencing data (e.g. edgeR, DEseq2). These approaches work by using all measured genes or loci to analyze and model the variance within and between replicates, thereby allowing the identification of genes/loci with significant differences between conditions.<br>A sample size of five mice were selected for xenograft experiments. A sample size of three biological replicates and two or three technical replicates was selected for cell growth assays. A sample size of two biological replicate experiments and two or three technical replicates was selected for qRT-PCR analyses. These sample sizes were selected to allow these experiments to be sufficiently powered such that differences between experimental groups will be statistically significant with type I error rates < 5% (e.g. $P < 0.05$) and with 80% power to detect a standardized difference. Wherever possible, additional biological replicates were included.
Data exclusions	All replicate data were used for statistical analysis and no data was excluded.
Replication	Three (for locus-specific perturbations) or fifteen (for multi-loci perturbations) recipient mice were performed for enCRISPRi perturbation screens. Two biological replicate screens were performed. Two biological replicate experiments and two or three technical replicates were performed for qRT-PCR analyses. Two biological replicates were performed for ChIP-seq and RNA-seq experiments. Replicate experiments yielded similar results.
Randomization	For xenograft experiments, recipient NSG mice were allocated randomly for the xenotransplantation of control or enCRISPRa expressing cells.
Blinding	Blinding was not possible for the xenograft and in vivo CRE perturbation experiments because we would need to the genotype or sample information for the downstream measurements.

Reporting for specific materials, systems and methods

We require information from authors about some types of materials, experimental systems and methods used in many studies. Here, indicate whether each material, system or method listed is relevant to your study. If you are not sure if a list item applies to your research, read the appropriate section before selecting a response.

Materials & experimental systems

n/a	Involved in the study	
[ ]	[x]	Antibodies
[ ]	[x]	Eukaryotic cell lines
[x]	[ ]	Palaeontology
[ ]	[x]	Animals and other organisms
[x]	[ ]	Human research participants
[x]	[ ]	Clinical data

Methods

n/a	Involved in the study	
[ ]	[x]	ChIP-seq
[ ]	[x]	Flow cytometry
[x]	[ ]	MRI-based neuroimaging

Antibodies

Antibodies used	H3K27ac, Abcam Cat# ab4729; RRID:AB_2118291<br>H3K4me1, Abcam Cat# ab8895; RRID:AB_306847<br>H3K4me2, Millipore Cat# 07-030 RRID:AB_10099880<br>H3K9me3, Abcam Cat# ab8898; RRID:AB_306848<br>H3K27me3, Millipore, Cat# 07-449 RRID:AB_310624<br>GATA1, Abcam Cat# ab11852; RRID:AB_298635<br>TAL1, Santa Cruz Biotechnology Cat# sc-12984; RRID:AB_2199699<br>CTCF, Millipore Cat# 07-729; RRID:AB_441965<br>HA, Santa Cruz Biotechnology Cat# sc-805; RRID:AB_631618<br>Cas9, Abcam Cat# ab191468 RRID:AB_2692325

	b-tubulin, Cell Signaling Technology, Cat#2128 RRID:AB_ 823664
Validation	All antibodies were commercially available, suitable for ChIP-seq and/or Western blot in human or mouse cells as specified by the manufacturers.

Eukaryotic cell lines

Policy information about cell lines

Cell line source(s)	Human Jurkat cell line, ATCC Cat# TIB-152; RRID:CVCL_0367<br>Human HEK293T cell line, ATCC Cat#CRL-3216; RRID:CVCL_0063:<br>Human K562 cell line, ATCC Cat# CCL-243; RRID:CVCL_0004
Authentication	To ensure the identity and validity of these cell lines, we perform qRT-PCR and Western Blot analyses of several leukemia signature genes (e.g. the 17-gene signature from Ng et al., 2016 Nature 540:433-437). We also performed RNA-seq and whole genome sequencing (WGS) and compared with the results in previous studies.
Mycoplasma contamination	All cell lines were tested negative for mycoplasma contamination.
Commonly misidentified lines (See ICLAC register)	No cell lines used in this study were found in the database of commonly misidentified cell lines that are maintained by ICLAC and NCBI BioSample.

Animals and other organisms

Policy information about studies involving animals; ARRIVE guidelines recommended for reporting animal research

Laboratory animals	NOD-SCID (NSG) mice were purchased from and maintained at the animal core facility of University of Texas Southwestern Medical Center (UTSW).
Wild animals	Study did not involve wild animals.
Field-collected samples	Study did not involve field-collected samples.
Ethics oversight	Animal work described in this manuscript has been approved and conducted under the oversight of the UT Southwestern Institutional Animal Care and Use Committee (IACUC) under the protocol APN 2017-102220.

Note that full information on the approval of the study protocol must also be provided in the manuscript.

ChIP-seq

Data deposition

- [x] Confirm that both raw and final processed data have been deposited in a public database such as GEO.
- [x] Confirm that you have deposited or provided access to graph files (e.g. BED files) for the called peaks.

Data access links<br>*May remain private before publication.*	https://www.ncbi.nlm.nih.gov/geo/query/acc.cgi?acc=GSE132216
Files in database submission	GSM3854054 ChIP-seq_Jurkat-H3K27ac<br>GSM3854055 ChIP-seq_293T_enCRISPRa_sgGal4_HA_rep1<br>GSM3854056 ChIP-seq_293T_enCRISPRa_sgGal4_HA_rep2<br>GSM3854057 ChIP-seq_293T_enCRISPRa_sgHS2_HA_rep1<br>GSM3854058 ChIP-seq_293T_enCRISPRa_sgHS2_HA_rep2<br>GSM3854059 ChIP-seq_K562_enCRISPRi-LK_sgGal4_HA_rep1<br>GSM3854060 ChIP-seq_K562_enCRISPRi-LK_sgGal4_HA_rep2<br>GSM3854061 ChIP-seq_K562_enCRISPRi-LK_sgHS2_HA_rep1<br>GSM3854062 ChIP-seq_K562_enCRISPRi-LK_sgHS2_HA_rep2<br>GSM3854063 ChIP-seq_K562_dCas9-KRAB_sgHS2_cas9_rep1<br>GSM3854064 ChIP-seq_K562_dCas9-KRAB_sgHS2_cas9_rep2<br>GSM3854065 ChIP-seq_K562_dCas9-KRAB_sgHS2_CTCF_rep1<br>GSM3854066 ChIP-seq_K562_dCas9-KRAB_sgHS2_CTCF_rep2<br>GSM3854067 ChIP-seq_K562_dCas9-KRAB_sgHS2_GATA1_rep1<br>GSM3854068 ChIP-seq_K562_dCas9-KRAB_sgHS2_GATA1_rep2<br>GSM3854069 ChIP-seq_K562_dCas9-KRAB_sgHS2_H3K27ac_rep1<br>GSM3854070 ChIP-seq_K562_dCas9-KRAB_sgHS2_H3K27ac_rep2<br>GSM3854071 ChIP-seq_K562_dCas9-KRAB_sgHS2_H3K4me2_rep1<br>GSM3854072 ChIP-seq_K562_dCas9-KRAB_sgHS2_H3K4me2_rep2<br>GSM3854073 ChIP-seq_K562_dCas9-KRAB_sgHS2_H3K4me_rep1<br>GSM3854074 ChIP-seq_K562_dCas9-KRAB_sgHS2_H3K4me_rep2

GSM3854075 ChIP-seq_K562_dCas9-KRAB_sgHS2_H3K9me3_rep1
GSM3854076 ChIP-seq_K562_dCas9-KRAB_sgHS2_H3K9me3_rep2
GSM3854077 ChIP-seq_K562_dCas9-KRAB_sgHS2_TAL1_rep1
GSM3854078 ChIP-seq_K562_dCas9-KRAB_sgHS2_TAL1_rep2
GSM3854079 ChIP-seq_K562_dCas9-LSD1_sgHS2_cas9_rep1
GSM3854080 ChIP-seq_K562_dCas9-LSD1_sgHS2_cas9_rep2
GSM3854081 ChIP-seq_K562_dCas9-LSD1_sgHS2_CTCF_rep1
GSM3854082 ChIP-seq_K562_dCas9-LSD1_sgHS2_CTCF_rep2
GSM3854083 ChIP-seq_K562_dCas9-LSD1_sgHS2_GATA1_rep1
GSM3854084 ChIP-seq_K562_dCas9-LSD1_sgHS2_GATA1_rep2
GSM3854085 ChIP-seq_K562_dCas9-LSD1_sgHS2_H3K27ac_rep1
GSM3854086 ChIP-seq_K562_dCas9-LSD1_sgHS2_H3K27ac_rep2
GSM3854087 ChIP-seq_K562_dCas9-LSD1_sgHS2_H3K4me2_rep1
GSM3854088 ChIP-seq_K562_dCas9-LSD1_sgHS2_H3K4me2_rep2
GSM3854089 ChIP-seq_K562_dCas9-LSD1_sgHS2_H3K4me_rep1
GSM3854090 ChIP-seq_K562_dCas9-LSD1_sgHS2_H3K4me_rep2
GSM3854091 ChIP-seq_K562_dCas9-LSD1_sgHS2_H3K9me3_rep1
GSM3854092 ChIP-seq_K562_dCas9-LSD1_sgHS2_H3K9me3_rep2
GSM3854093 ChIP-seq_K562_dCas9-LSD1_sgHS2_TAL1_rep1
GSM3854094 ChIP-seq_K562_dCas9-LSD1_sgHS2_TAL1_rep2
GSM3854095 ChIP-seq_K562_enCRISPRi-KL_sgHS2_cas9_rep1
GSM3854096 ChIP-seq_K562_enCRISPRi-KL_sgHS2_cas9_rep2
GSM3854097 ChIP-seq_K562_enCRISPRi-KL_sgHS2_CTCF_rep1
GSM3854098 ChIP-seq_K562_enCRISPRi-KL_sgHS2_CTCF_rep2
GSM3854099 ChIP-seq_K562_enCRISPRi-KL_sgHS2_GATA1_rep1
GSM3854100 ChIP-seq_K562_enCRISPRi-KL_sgHS2_GATA1_rep2
GSM3854101 ChIP-seq_K562_enCRISPRi-KL_sgHS2_H3K27ac_rep1
GSM3854102 ChIP-seq_K562_enCRISPRi-KL_sgHS2_H3K27ac_rep2
GSM3854103 ChIP-seq_K562_enCRISPRi-KL_sgHS2_H3K4me2_rep1
GSM3854104 ChIP-seq_K562_enCRISPRi-KL_sgHS2_H3K4me2_rep2
GSM3854105 ChIP-seq_K562_enCRISPRi-KL_sgHS2_H3K4me_rep1
GSM3854106 ChIP-seq_K562_enCRISPRi-KL_sgHS2_H3K4me_rep2
GSM3854107 ChIP-seq_K562_enCRISPRi-KL_sgHS2_H3K9me3_rep1
GSM3854108 ChIP-seq_K562_enCRISPRi-KL_sgHS2_H3K9me3_rep2
GSM3854109 ChIP-seq_K562_enCRISPRi-KL_sgHS2_TAL1_rep1
GSM3854110 ChIP-seq_K562_enCRISPRi-KL_sgHS2_TAL1_rep2
GSM3854111 ChIP-seq_K562_enCRISPRi-LK_sgHS2_cas9_rep1
GSM3854112 ChIP-seq_K562_enCRISPRi-LK_sgHS2_cas9_rep2
GSM3854113 ChIP-seq_K562_enCRISPRi-LK_sgHS2_CTCF_rep1
GSM3854114 ChIP-seq_K562_enCRISPRi-LK_sgHS2_CTCF_rep2
GSM3854115 ChIP-seq_K562_enCRISPRi-LK_sgHS2_GATA1_rep1
GSM3854116 ChIP-seq_K562_enCRISPRi-LK_sgHS2_GATA1_rep2
GSM3854117 ChIP-seq_K562_enCRISPRi-LK_sgHS2_H3K27ac_rep1
GSM3854118 ChIP-seq_K562_enCRISPRi-LK_sgHS2_H3K27ac_rep2
GSM3854119 ChIP-seq_K562_enCRISPRi-LK_sgHS2_H3K4me2_rep1
GSM3854120 ChIP-seq_K562_enCRISPRi-LK_sgHS2_H3K4me2_rep2
GSM3854121 ChIP-seq_K562_enCRISPRi-LK_sgHS2_H3K4me_rep1
GSM3854122 ChIP-seq_K562_enCRISPRi-LK_sgHS2_H3K4me_rep2
GSM3854123 ChIP-seq_K562_enCRISPRi-LK_sgHS2_H3K9me3_rep1
GSM3854124 ChIP-seq_K562_enCRISPRi-LK_sgHS2_H3K9me3_rep2
GSM3854125 ChIP-seq_K562_enCRISPRi-LK_sgHS2_TAL1_rep1
GSM3854126 ChIP-seq_K562_enCRISPRi-LK_sgHS2_TAL1_rep2
GSM3854127 ChIP-seq_K562_sgGal4_Cas9_rep1
GSM3854128 ChIP-seq_K562_sgGal4_Cas9_rep2
GSM3854129 ChIP-seq_K562_sgGal4_CTCF_rep1
GSM3854130 ChIP-seq_K562_sgGal4_CTCF_rep2
GSM3854131 ChIP-seq_K562_sgGal4_GATA1_rep1
GSM3854132 ChIP-seq_K562_sgGal4_GATA1_rep2
GSM3854133 ChIP-seq_K562_sgGal4_H3K27ac_rep1
GSM3854134 ChIP-seq_K562_sgGal4_H3K27ac_rep2
GSM3854135 ChIP-seq_K562_sgGal4_H3K4me2_rep1
GSM3854136 ChIP-seq_K562_sgGal4_H3K4me2_rep2
GSM3854137 ChIP-seq_K562_sgGal4_H3K4me_rep1
GSM3854138 ChIP-seq_K562_sgGal4_H3K4me_rep2
GSM3854139 ChIP-seq_K562_sgGal4_H3K9me3_rep1
GSM3854140 ChIP-seq_K562_sgGal4_H3K9me3_rep2
GSM3854141 ChIP-seq_K562_sgGal4_TAL1_rep1
GSM3854142 ChIP-seq_K562_sgGal4_TAL1_rep2

GSM4190316 ChIP-seq_K562_dCas9-KRAB_sgHS2_H3K27me3_rep1
GSM4190317 ChIP-seq_K562_dCas9-KRAB_sgHS2_H3K27me3_rep2
GSM4190318 ChIP-seq_K562_dCas9-LSD1_sgHS2_H3K27me3_rep1
GSM4190319 ChIP-seq_K562_dCas9-LSD1_sgHS2_H3K27me3_rep2
GSM4190320 ChIP-seq_K562_enCRISPRi-KL_sgHS2_H3K27me3_rep1
GSM4190321 ChIP-seq_K562_enCRISPRi-KL_sgHS2_H3K27me3_rep2
GSM4190322 ChIP-seq_K562_enCRISPRi-LK_sgHS2_H3K27me3_rep1
GSM4190323 ChIP-seq_K562_enCRISPRi-LK_sgHS2_H3K27me3_rep2
GSM4190324 ChIP-seq_K562_sgGal4_H3K27me3_rep1
GSM4190325 ChIP-seq_K562_sgGal4_H3K27me3_rep2
GSM3854143 RNA-seq_K562_dCas9-KRAB_sgHS2_rep1
GSM3854144 RNA-seq_K562_dCas9-KRAB_sgHS2_rep2
GSM3854145 RNA-seq_K562_dCas9-LSD1_sgHS2_rep1
GSM3854146 RNA-seq_K562_dCas9-LSD1_sgHS2_rep2
GSM3854147 RNA-seq_K562_enCRISPRi-KL_sgHS2_rep1
GSM3854148 RNA-seq_K562_enCRISPRi-KL_sgHS2_rep2
GSM3854149 RNA-seq_K562_enCRISPRi-LK_sgHS2_rep1
GSM3854150 RNA-seq_K562_enCRISPRi-LK_sgHS2_rep2
GSM3854151 RNA-seq_K562_sgGal4_rep1
GSM3854152 RNA-seq_K562_sgGal4_rep2
GSM3854041 ATAC-seq_Jurkat

Genome browser session (e.g. UCSC)

N/A

Methodology

Replicates

See Supplementary Table 1. Two replicate experiments were performed for ChIP-seq experiments following enCRISPRi. The replicates closely agree when assessed by peak overlap, clustering analysis with input control or ChIP-seq data for other factors, or motif enrichment analysis.

Sequencing depth

See detailed information of the deposited datasets in GEO (accession #GSE132216).

Antibodies

H3K27ac, Abcam Cat# ab4729; RRID:AB_2118291
H3K4me1, Abcam Cat# ab8895; RRID:AB_306847
H3K4me2, Millipore Cat# 07-030 RRID:AB_10099880
H3K9me3, Abcam Cat# ab8898; RRID:AB_306848
H3K27me3, Millipore, Cat# 07-449 RRID:AB_310624
GATA1, Abcam Cat# ab11852; RRID:AB_298635
TAL1, Santa Cruz Biotechnology Cat# sc-12984; RRID:AB_2199699
CTCF, Millipore Cat# 07-729; RRID:AB_441965
HA, Santa Cruz Biotechnology Cat# sc-805; RRID:AB_631618
Cas9, Abcam Cat# ab191468 RRID:AB_2692325

Peak calling parameters

Peaks were identified by MACS version 2 with P value of 10e-5, and ranked by fold enrichment and P value in each dataset.

Data quality

Peaks were called at a P value of 10e-5. The replicate experiments closely agree when assessed by peak overlap, clustering analysis with input control or ChIP-seq data for other factors, or motif enrichment analysis.

Software

ChIP-seq raw reads were aligned to the human (hg19) genome assembly using Bowtie2 with –k 1. Only tags that uniquely mapped to the genome were used for further analysis. Peaks were identified by MACS version 2 with P value of 10e-5, and ranked by fold enrichment and P value in each dataset.

Flow Cytometry

Plots

Confirm that:

- [x] The axis labels state the marker and fluorochrome used (e.g. CD4-FITC).
- [x] The axis scales are clearly visible. Include numbers along axes only for bottom left plot of group (a 'group' is an analysis of identical markers).
- [x] All plots are contour plots with outliers or pseudocolor plots.
- [x] A numerical value for number of cells or percentage (with statistics) is provided.

Methodology

Sample preparation

BM cells were obtained by crushing femurs, tibias, vertebrae and pelvic bones with a mortar in Ca2+ and Mg2+-free Hank's buffered salt solution (HBSS, Gibco) supplemented with 2% heat-inactivated bovine serum (HIBS, Gibco). Spleens were

	dissociated by crushing followed by trituration. All BM and spleen cell suspensions were filtered through 70 µm cell strainers, followed by cell counting using a Vi-CELL cell viability analyser (Beckman Coulter).
Instrument	FACSAria or FACSCanto flow cytometer (BD Biosciences)
Software	FACSDiva (BD Biosciences)
Cell population abundance	Variable between 10~50% depending on the cell populations (e.g. myeloid, T, or B lymphoid cells)
Gating strategy	Cells were incubated with combinations of fluorophore-conjugated antibodies. Lineage markers for HSCs and progenitors were CD2, CD3, CD5, CD8, B220, Gr1 and Ter119. Antibody staining was performed at 4°C for 30min or on ice for 90min. Biotinylated antibodies were visualized by incubation with PE/Cy7-conjugated streptavidin at 4°C for 30min. DAPI (4,6-diamidino-2-phenylindole; 2µg/ml in PBS) was used to exclude dead cells.

☒ Tick this box to confirm that a figure exemplifying the gating strategy is provided in the Supplementary Information.
